# Role of intestinal trefoil factor in protecting intestinal epithelial cells from burn-induced injury

**DOI:** 10.1038/s41598-018-21282-4

**Published:** 2018-02-16

**Authors:** Jianhong Hu, Yan Shi, Chao Wang, Hanxing Wan, Dan Wu, Hongyu Wang, Xi Peng

**Affiliations:** Institute of Burn Research, State Key Laboratory of Trauma, Burns and Combined Injury, Southwest Hospital, The Third Military Medical University, Chongqing, 400038 China

## Abstract

Although intestinal trefoil factor (ITF) can alleviate the burn-induced intestinal mucosa injury, the underlying mechanisms remains elusive. In this study, we investigated if ITF alters glutamine transport on the brush border membrane vesicles (BBMVs) of the intestines in Sprague-Dawley rats inflicted with 30% TBSA and the underlying mechanisms. We found that ITF significantly stimulated intestinal glutamine transport in burned rats. Mechanistically, ITF enhanced autophagy, reduces endoplasmic reticulum stress (ERS), and alleviates the impaired PDI, ASCT2, and B0AT1 in IECs and BBMVs after burn injury likely through AMPK activation. Therefore, ITF may protect intestinal epithelial cells from burn-induced injury through improving glutamine transport by alleviating ERS.

## Introduction

Extensive severe burns not only lead to major damage of the skin and subcutaneous tissues, but also cause varying degrees of multiple organ injuries^1^. One of the most seriously postburn-induced injury of organs is the intestine, mainly due to the hypoxia-caused ischemia. In this setting, abnormal energy metabolism aggravates intestinal mucosal injury and restrains mucosal repair as well^2,3^.

Glutamine (Gln) is an important energy source for maintaining the structure and function of human intestinal mucosa^4,5^. A series of studies have confirmed that Gln administration can significantly improve intestinal energy metabolism and promote ATP production in the postburn animals and patients, thereby alleviating intestinal mucosal injury and promoting mucosal repair^6–9^. Therefore, many treatment guidelines for critical care and severe burns recommend the routine use of Gln preparations to the patients^10,11^. The transport of Gln from the intestinal lumen to intestinal epithelial cells (IECs) is a crucial initial step in Gln metabolism in the intestine. Many studies showed that intestinal Gln transport is decreased significantly in abdominal trauma, systemic infections, and sepsis^12–14^. However, the underlying mechanisms remain unclear. Our previous work also found that enteral administration of Gln to the burn patients could not remarkably increase the intestinal transport of Gln compared to healthy subjects^15^, suggesting that burn-induced injury of the intestine could compromise its abilities to transport and utilize Gln.

There are several specific Gln transporters on the brush border of IECs, among which the sodium-dependent amino acid transporters ASCT2 and B0AT1 stand out^16,17^. ASCT2 and B0AT1 are special membrane transporters that depend on precise post-translational modification and folding in the endoplasmic reticulum (ER) for proper functioning^18^. Related studies have found that N-terminal glycosylation of ASCT2 in the ER after synthesis is key to its function^19–22^. Numerous studies have shown that severe endoplasmic reticulum stress (ERS) occurs in multiple organs after severe burns, resulting in ER dysfunction and impaired post-translational modifications^23^. We speculate that ERS may affect post-translational modification and folding for proper function of ASCT2 and B0AT1 in the ER. Therefore, the present study was undertaken to address these issues.

A variety of drugs can improve intestinal Gln transport under some pathological conditions. For example, growth hormone can improve Gln utilization in the patients with short bowel syndrome by augmenting the protein level of Gln transporters^24^. Epidermal growth factor (EGF) can effectively boost the protein level of ASCT2 and Gln transport in intestinal ischemia^13^. Insulin-like growth factor-I(IGF-I) can stimulate sodium-dependent Gln transport in IECs in piglets^25^. Our previous study also revealed that intestinal trefoil factor (ITF), an intestine-specific growth factor, can significantly improve the bioavailability of Gln when administered intragastrically to the burned animals^26^. However, it remains unclear whether ITF exerts this effect by modifying intestinal Gln transporters though ERS alleviation in IECs after burn injury.

ITF secreted by intestinal goblet cells can stabilize intestinal mucosa due to its special spatial structure^27,28^. Recent studies revealed that ITF can maintain intestinal mucosal integrity and promote cell proliferation and migration of IECs^29,30^. Loss of TFF1, a member of the trefoil peptide family, caused significant morphologic changes in the ER^31^. Since ITF and TFF1 are the members of same protein family, we wonder if loss of ITF may also alter the ER in IECs. Therefore, in the present study we investigated whether ITF can promote the expression and function of ASCT2 and B0AT1 proteins after burn injury; and if so, what are the underlying molecular mechanisms.

## Results

### Effect of ITF on the burn-induced change in Gln transport by BBMVs of IECs

**Figure 1:**
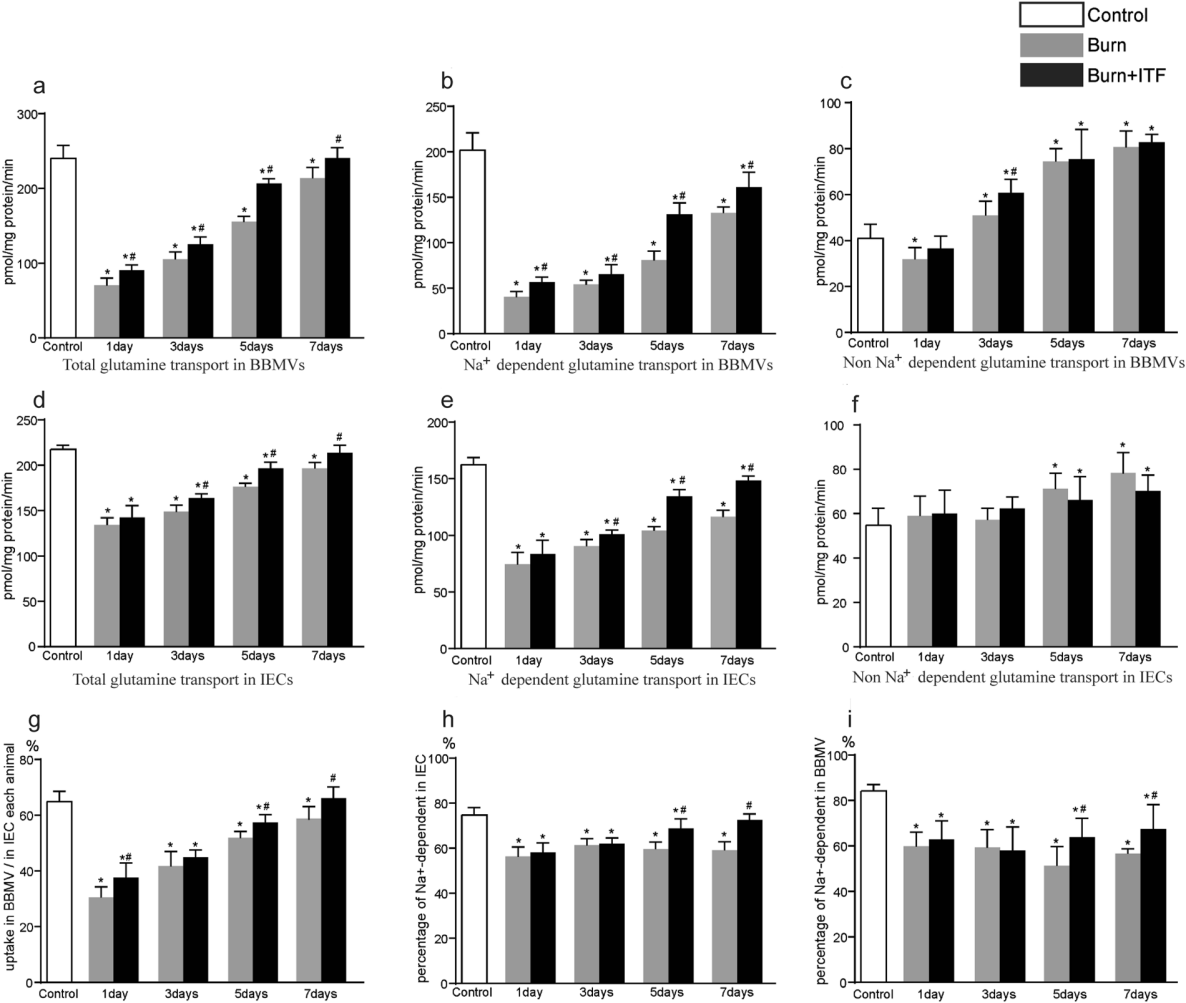
Effect of ITF on the burn-induced change in Gln transport by BBMVs of IECs. (**a**) Total glutamine transport in BBMVs, (**b**) Na^+^ dependent glutamine transport in BBMVs, (**c**) Na^+^ independent glutamine transport in BBMVs, (**d**) Total glutamine transport in IECs, (**e**) Na^+^ dependent glutamine transport in IECs, (**f**) Non Na^+^ independent glutamine transport in IECs, (**g**) Percentage of glutamine transport in BBMVs to in IECs of each animal, (**h**) Percentage of Na^+^ dependent glutamine transport in IECs, (**i**) Percentage of Na^+^ dependent glutamine transport in BBMVs. Datas were presented as means ± SD and analyzed by two-way ANOVAs. ^*^P < 0.05, compared to control group animals; ^#^*P* < 0.05, compared to burn group animals.

The results showed that the total transport abilities of Gln in BBMVs and IECs were significantly reduced after burn injury (*P* < 0.05) (Fig. 1a,d), and that sodium-dependent Gln transport was especially attenuated (*P* < 0.05) (Fig. 1b,e). The uptake of Gln by this transporter in BBMVs was decreased approximately 80% on day 1 and was still markedly lower on day 7 after burn injury (*P* < 0.05) (Fig. 1b).

In contrast, non-sodium-dependent Gln transport was increased after the injury except on day 1 (*P* < 0.05) (Fig. 1c,f). Although the absorption of Gln mediated by BBMVs was 65% of the total Gln transport by IECs before burn, this value was declined sharply on day 1 and was still markedly lower on day 7 after burn (*P* < 0.05) (Fig. 1g). The proportion of Gln transport mediated by the sodium-dependent Gln transporter was decreased to 60% after burn injury, approximately 30% decline (P < 0.05) (Fig. 1h,i).

ITF significantly improved total Gln transport in IECs and BBMVs on days 3 to 7 after injury (*P* < 0.05) (Fig. 1a,d). ITF enhanced the activity of the sodium-dependent Gln transporter and therefore increased the proportion of Gln transport in IECs and BBMVs (*P* < 0.05) after burn injury (Fig. 1h,i) but not the non-sodium-dependent transporter.

### Effect of ITF on the burn-induced morphological change in BBMVs of IECs

**Figure 2:**
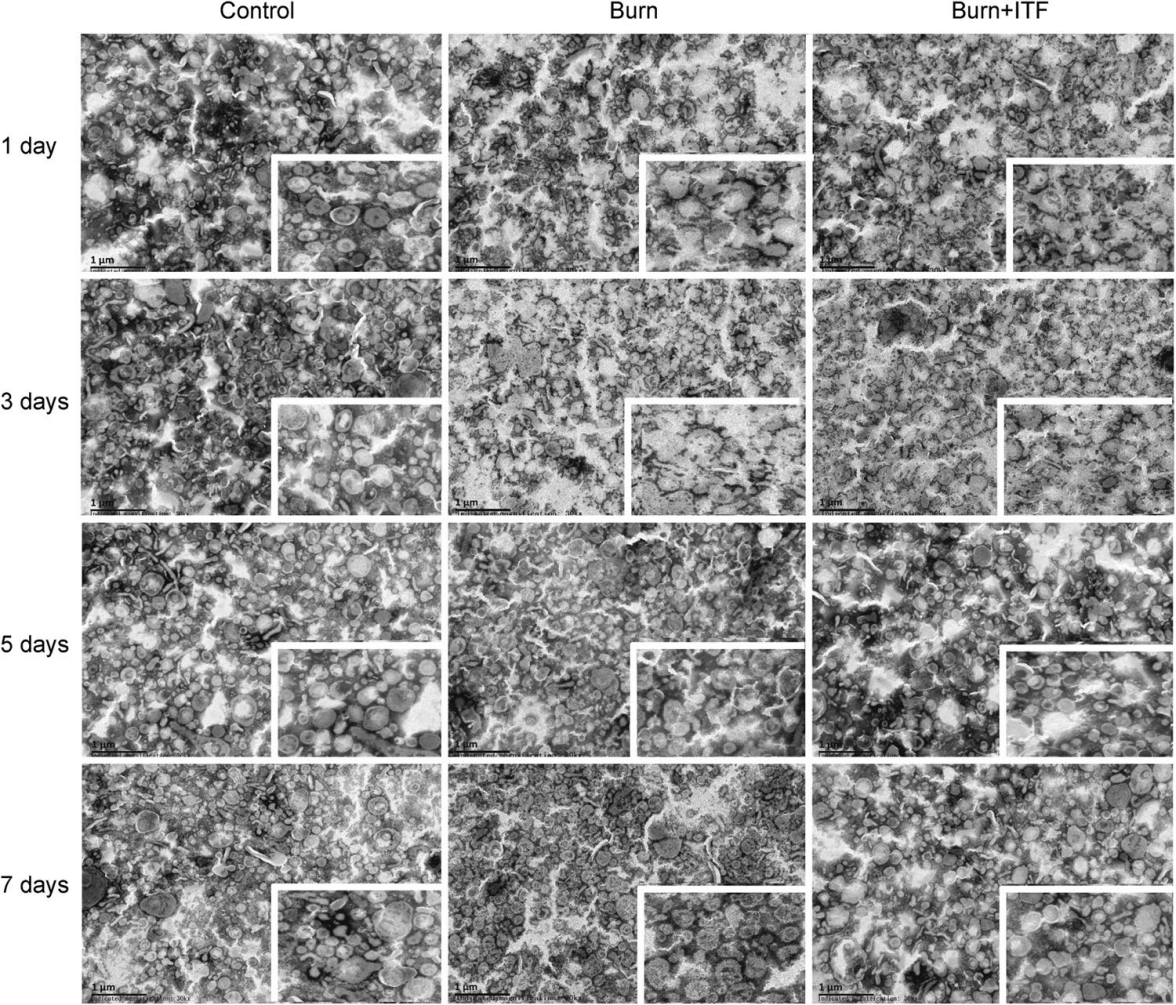
Effect of ITF on the burn-induced morphological change in BBMVs of IECs. The morphological structure of BBMV was observed by transmission electron microscope (bar, 1 μm) (n = 10/group per time point).

Using electron microscopy, we observed that BBMVs were structurally impaired in the intestine of burned rats. On days 1 to 3 after burn injury, the spatial structure of BBMVs was severely damaged, as manifested by the irregular shapes, broken vesicles, collapsed vesicle walls, rough borders, and many broken vesicle pieces. On days 5 to 7 after burn, the structure of BBMVs was gradually recovered with relatively clearly defined contours, but vesicle walls remained collapsed and borders remained rough (Fig. 2).

ITF significantly improved the burn-induced morphological changes in BBMVs, which returned to almost normal on day 7 after burn injury with intact vesicle membrane structure, round vesicles, and clearly defined contours, but the borders remained rough (Fig. 2). Therefore, ITF can rescue the integrity of BBMVs after burn injury.

### Effect of ITF on the burn-induced changes in the expression of ASCT2 and B0AT1 in IECs

**Figure 3:**
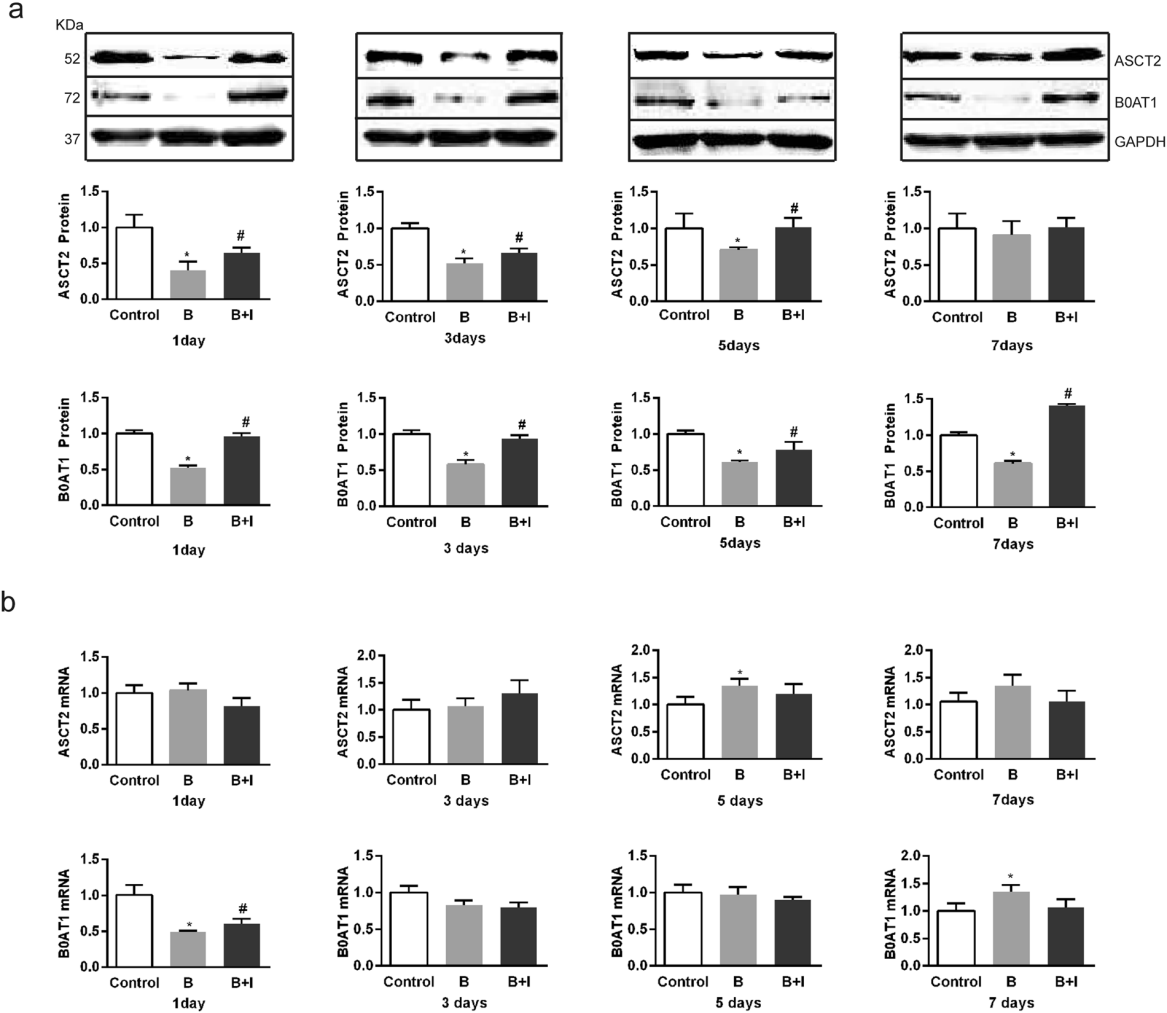
Effect of ITF on the burn-induced changes in the expression of ASCT2 and B0AT1 in IECs. (**a**) The protein levels of ASCT2 and B0AT1 were respectively detected by western bloting analysis (n = 10/group per time point). (**b**) The mRNA levels of ASCT2 and B0AT1 were respectively detected by Q-PCR (n = 10/group per time point). Datas were presented as means ± SD and analyzed two-way ANOVAs. ^*^P < 0.05, compared to control group animals; ^#^*P* < 0.05, compared to burn group animals.

Since ASCT2 and B0AT1 constitute main sodium-dependent Gln transporters in the intestine^16,17^, it is logical to test their involvements. We found that their protein expression levels in IECs were decreased markedly on days 1 to 7 after burn injury (*P* < 0.05) (Fig. 3a). However, ITF significantly enhanced the protein expression levels of ASCT2 and B0AT1, both returning to almost normal levels on day 7 after burn (Fig. 3a). Nonetheless, the mRNA level of ASCT2 in IECs was increased on day 5 (Fig. 3b), and the mRNA level of B0AT1 was decreased markedly only on day 1 after burn (*P* < 0.05) (Fig. 3b). ITF did not significantly alter the mRNA levels of ASCT2 and B0AT1, except rescuing B0AT1 mRNA on day 1 only after burn (*P* < 0.05) (Fig. 3b). Therefore, ITF can rescue the protein but not mRNA expression levels of ASCT2 and B0AT1 in the intestine after burn injury.

### Effect of ITF on the burn-induced change in ERS indicators of IECs

**Figure 4:**
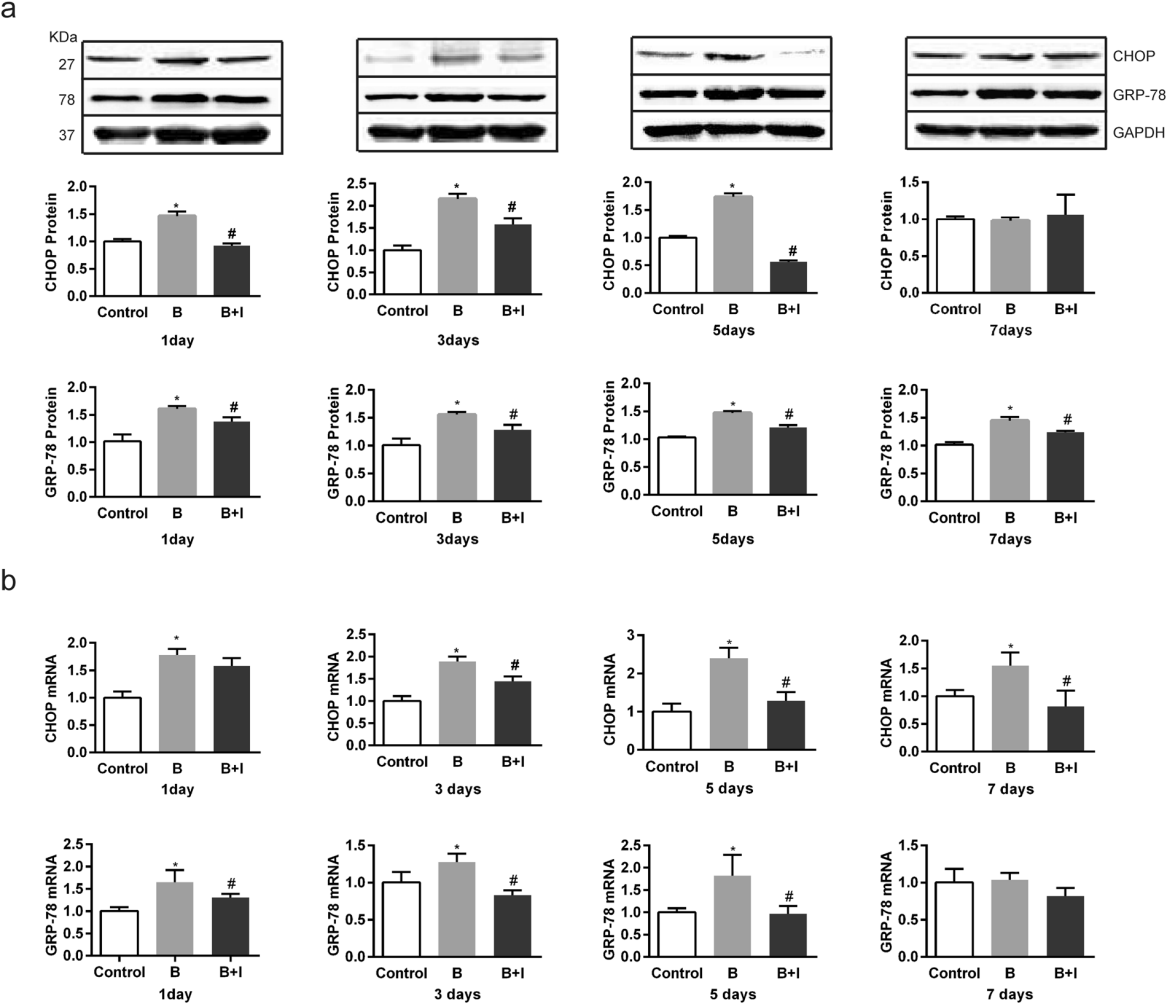
Effect of ITF on the burn-induced change in ERS indicators of IECs. IECs of Sprague-dawley rats were lysed for western bloting analysis of ERS indicator (**a**) CHOP and GRP-78 protein levels, (**b**) CHOP and GRP-78 mRNA levels) (n = 10/group per time point). Datas were presented as means ± SD and analyzed by two-way ANOVAs. ^*^P < 0.05, compared to control group animals; ^#^*P* < 0.05, compared to burn group animals.

Since extensive ERS occurs in the cells of several organs secondary to severe burns^23^, we tested the involvements of CHOP and GRP-78, ERS marker proteins. CHOP protein in IECs was increased significantly on days 1 to 5 after burn injury, but dropped to the baseline on day 7 (*P* < 0.05) (Fig. 4a). GRP-78 protein was also increased significantly on days 1 to 7 after burn injury (*P* < 0.05) (Fig. 4a). The mRNA levels of CHOP and GRP-78 share similar patterns with their protein levels (Fig. 4b). ITF markedly decreased both protein and mRNA levels of CHOP and GRP-78 (*P* < 0.05) (Fig. 4), indicating that ITF can alleviate burn-induced ERS in IECs.

### Effect of ITF on the burn-induced change in protein disulfide isomerase in IECs

**Figure 5:**
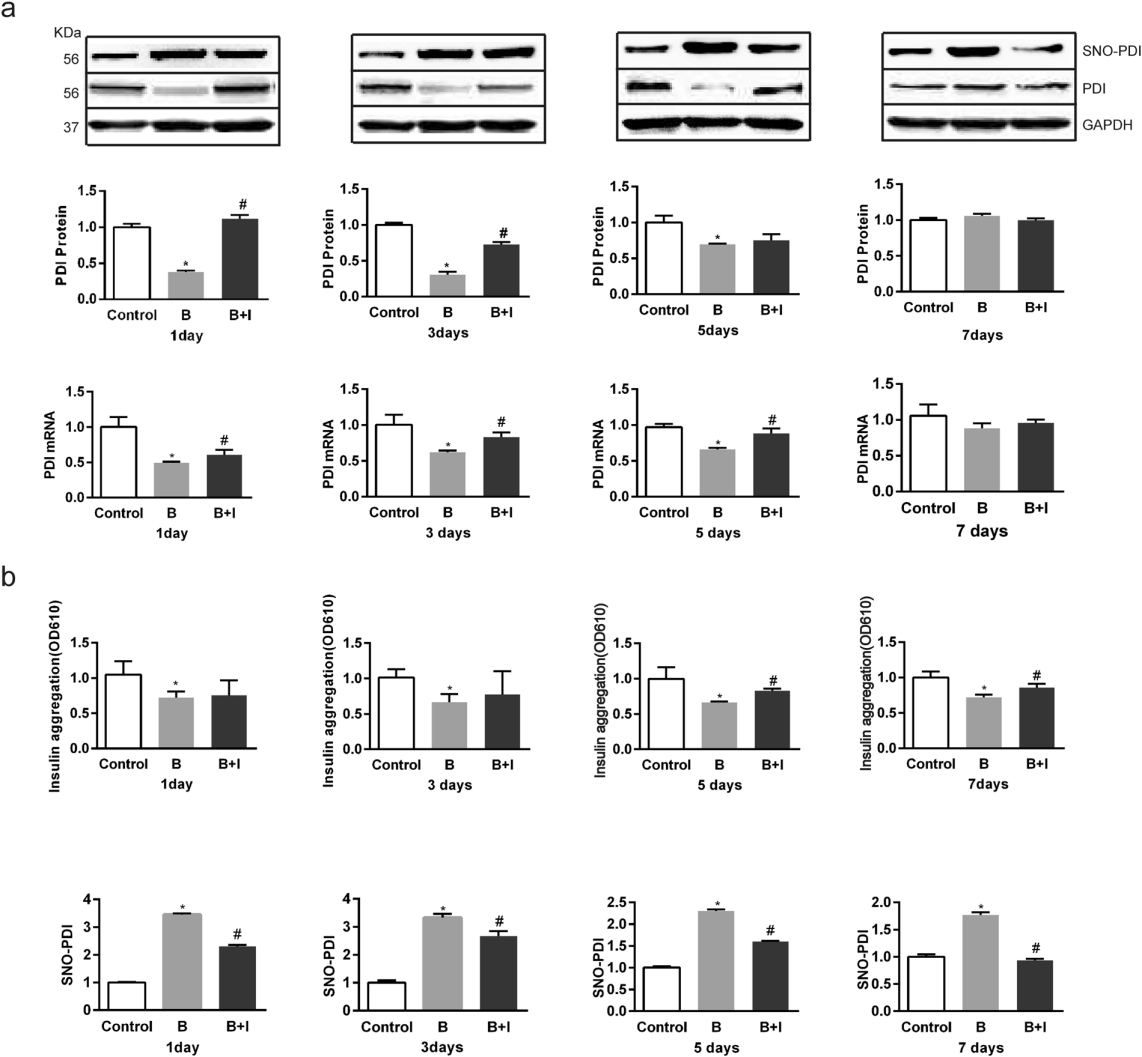
Effect of ITF on the burn-induced change in protein disulfide isomerase in IECs. (**a**) The protein level of PDI was respectively detected by western bloting analysis (n = 10/group per time point), the mRNA level of PDI was respectively detected by Q-PCR (n = 10/group per time point). (**b**) PDI activity was measured using an insulin aggregation assay or detecting SNO-PDI protein level by western bloting analysis. Datas were presented as means ± SD and analyzed two-way ANOVAs. ^*^P < 0.05, compared to control group animals; ^#^*P* < 0.05, compared to burn group animals.

Extensive ERS affects the expression and activity of protein disulfide isomerase (PDI), an ER chaperone and protein-folding catalyst^32,33^. We found both mRNA and protein expression levels of PDI were decreased significantly but almost recovered to the baseline on day 7 after burn injury (Fig. 5a). ITF markedly enhanced the PDI protein and mRNA levels (Fig. 5a). We also studied the PDI activity with an insulin aggregation assay, and found that the PDI activity in IECs was significantly decreased on days 1 to 7 after burn injury (Fig. 5b). ITF markedly heightened the PDI activity on day 5, suggesting that ITF can enhance both expression and activity of PDI in IECs after burn injury.

NO-mediated S-nitrosylation of PDI (SNO-PDI) can inhibit PDI activity and its beneficial effects^34^. We found SNO-PDI level in IECs was significantly increased on days 1 to 7 after burn injury (Fig. 5b). ITF markedly reduced the SNO-PDI level (Fig. 5b), further supporting our notion that ITF can rescue PDI in IECs after burn injury.

### Effect of ITF on the burn-induced changes in AMPK phosphorylation and autophagy in rat IECs

**Figure 6:**
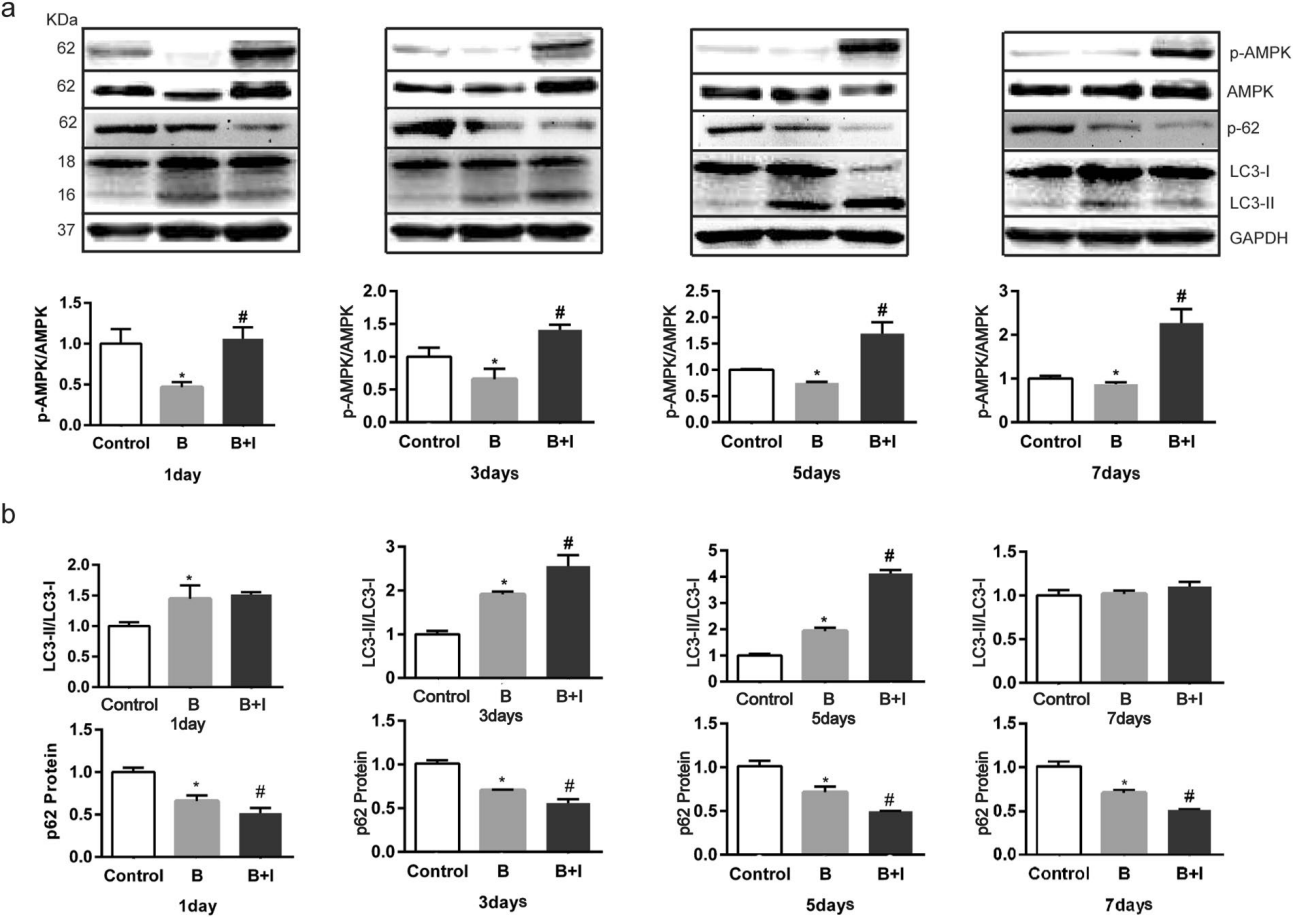
Effect of ITF on the burn-induced changes in AMPK phosphorylation and autophagy in rat IECs. (**a**) The AMPK phosphorylation level (p-AMPK/AMPM) was detected by western bloting (n = 10/group per time point). (**b**) Autophagy levels (LC3-II/LC3-I, p-62) were detected by western bloting (n = 10/group per time point). Datas were presented as means ± SD and analyzed by two-way ANOVAs. ^*^P < 0.05, compared to control group animals; ^#^*P* < 0.05, compared to burn group animals.

AMPK is an important upstream regulator of autophagy, and its phosphorylation promotes autophagy^35–37^. The present study showed that 1 to 5 days after burn injury, the AMPK phosphorylation level decreased significantly (*P* < 0.05) (Fig. 6a). Compared with the burn group, ITF administration significantly improved the level of AMPK phosphorylation, with statistical significance 1 to 7 days after ITF administration. The LC3-II/LC3-I ratio, an autophagy marker, increased significantly (*P* < 0.05) (Fig. 6b). Moreover, we also investigated another autophagy indicator, the p62 protein level, and we found that the p62 protein level in rat IECs obviously decreased 1 to 7 days after bury injury (*P* < 0.05) (Fig. 6b). Compared with the burn group, the increase in the LC3-II/LC3-I ratio was more pronounced 3 to 5 days after ITF administration, and ITF markedly boosted the reduction of the p62 protein level (*P* < 0.05) (Fig. 6b). These results suggested that ITF can improve the AMPK phosphorylation level and enhance autophagy in rat IECs after burn injury.

### PDI inactivation on the biological activity of ITF

**Figure 7:**
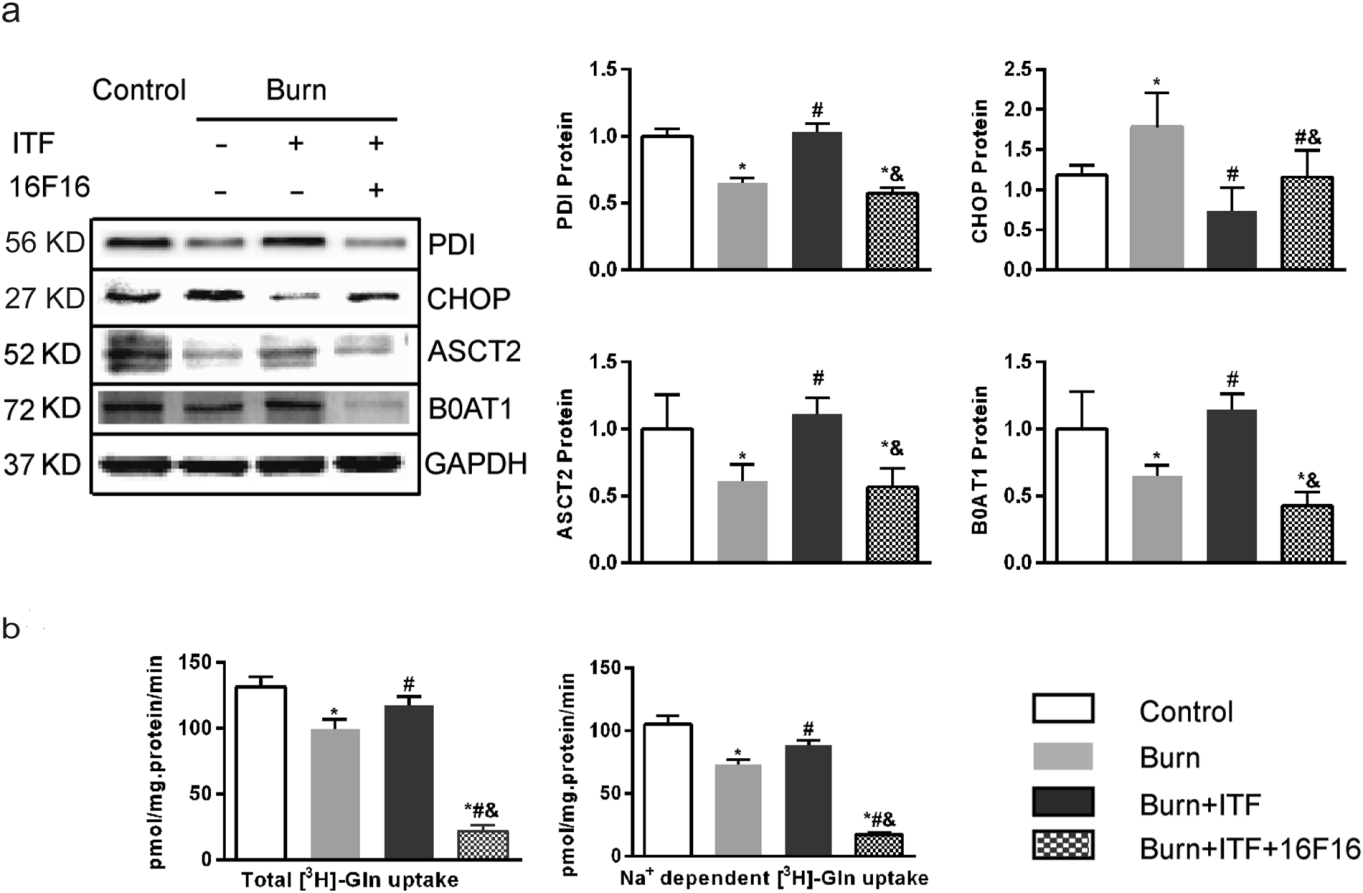
PDI inactivation on the biological activity of ITF (**a**) Western blotting analysis for protein levels of PDI, CHOP, ASCT2, B0AT1. (**b**) Variation of glutamine transport in IEC-6 cells under different treatment factor. Datas were presented as means ± SD and analyzed by two-way ANOVAs. ^*^P < 0.05, compared to control; ^#^*P* < 0.05, compared to burned serum group; ^&^*P* < 0.05, compared to burned serum and ITF treated cells.

To verify whether PDI has an important role in the mechanism by which ITF promotes the protein levels of ASCT2 and B0AT1 and improves Gln transport, this study applied 16F16, a PDI inhibitor^38^, to observe changes in the biological activity of ITF after PDI suppression. The results showed that after IEC-6 cells were co-cultured with burn serum for 12 hours, the protein levels of PDI, ASCT2, and B0AT1 decreased significantly (*P* < 0.05) (Fig. 7a), the CHOP protein level increased markedly (*P* < 0.05) (Fig. 7a), while Gln transport by IEC-6 decreased substantially (*P* < 0.05) (Fig. 7b). Compared with the burn serum group, ITF administration significantly reduced the protein level of CHOP (*P* < 0.05) (Fig. 7a), promoted the protein levels of PDI, ASCT2, and B0AT1 (*P* < 0.05) (Fig. 7a), and enhanced Gln transport by IEC-6 (*P* < 0.05) (Fig. 7b).

Compared with ITF treatment, after administration of 16F16 (10 μmol/L), the PDI protein level decreased enormously (*P* < 0.05) (Fig. 7a), the CHOP protein level increased sharply (*P* < 0.05) (Fig. 7a), while the ASCT2 and B0AT1 protein level decreased significantly and Gln transport by IEC-6 decreased (*P* < 0.05) (Fig. 7a,b). The results of this study showed that after the suppression of PDI, ERS was enhanced, the protein level of Gln transporters was reduced, and Gln transport by IEC-6 was impaired, which indicates that ITF indeed improves intestinal Gln transport by upregulating the protein level of PDI and promoting the protein levels of ASCT2 and B0AT1.

### Inhibition of autophagy mitigates the biological effect of ITF on IEC-6 cells

**Figure 8:**
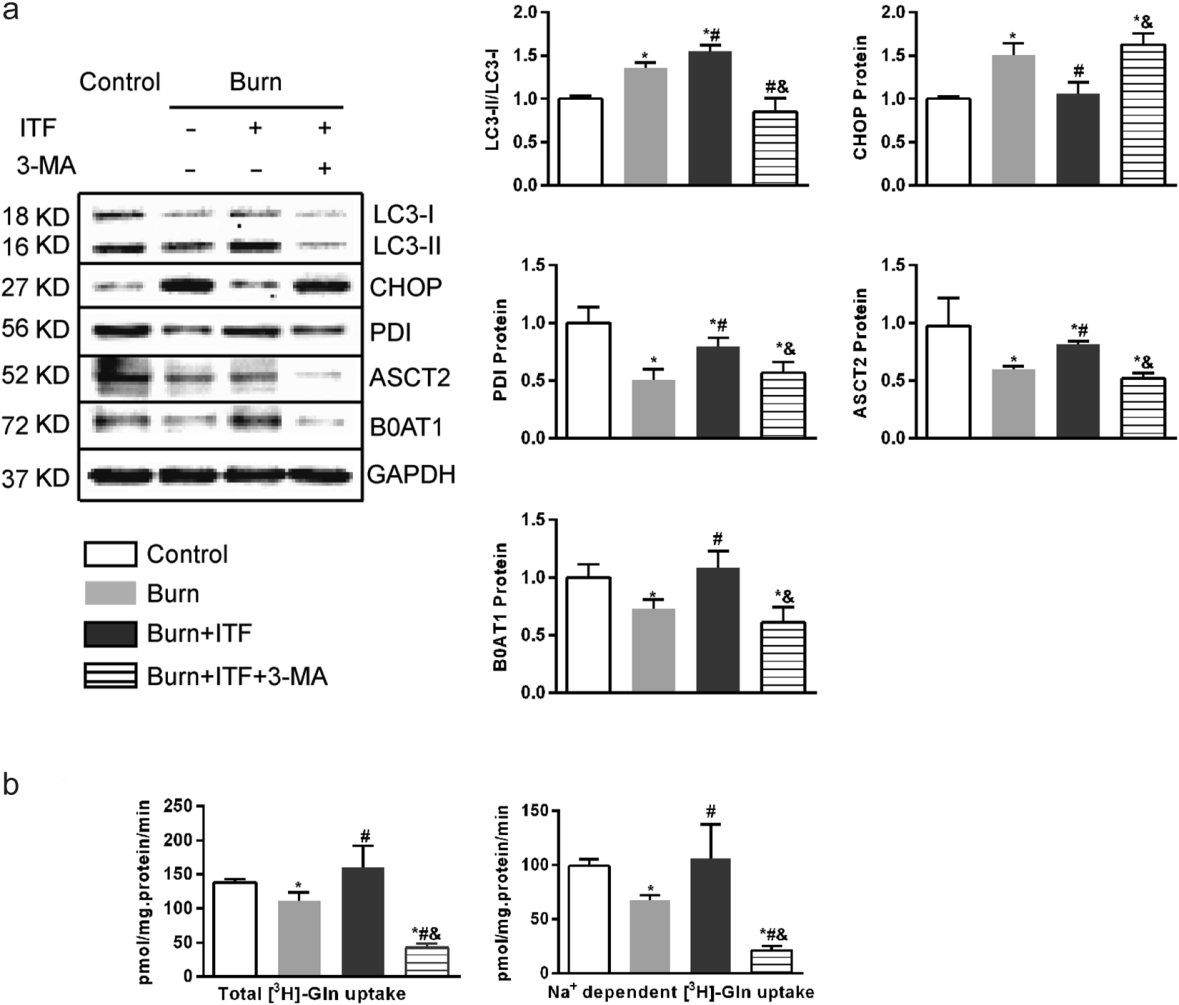
Inhibition of autophagy mitigates the biological effect of ITF on IEC-6 cells. (**a**) Western blotting analysis for protein levels of LC3-II, CHOP, PDI, ASCT2, B0AT1. (**b**) Variation of glutamine transport in IEC-6 cells under different treatment factor. Datas were presented as means ± SD and analyzed by two-way ANOVAs. ^*^P < 0.05, compared to control; ^#^*P* < 0.05, compared to burned serum group; ^&^*P* < 0.05, compared to burned serum and ITF treated cells.

It is known that autophagy can alleviate ERS by removing abnormal proteins^39,40^. To confirm whether the regulation of autophagy is a key step in the mechanism by which ITF alleviates post-burn ERS and increases the protein levels of PDI, ASCT2, and B0AT1, this study applied 3-methyl adenine (3-MA), an autophagy inhibitor, to observe changes in the biological activity of ITF. The results showed that after IEC-6 cells were co-cultured with burn serum for 12 hours, the protein levels of PDI, ASCT2, and B0AT1 decreased significantly (*P* < 0.05) (Fig. 8a), the CHOP protein level increased markedly (*P* < 0.05) (Fig. 8a), the LC3-II/LC3-I ratio increased (*P* < 0.05) (Fig. 8a), while Gln transport by IEC-6 decreased substantially (*P* < 0.05) (Fig. 8b). Compared with the burn serum group, ITF administration significantly increased the LC3-II/LC3-I ratio sharply (*P* < 0.05) (Fig. 8a), reduced the protein level of CHOP (*P* < 0.05) (Fig. 8a), promoted the protein levels of PDI, ASCT2, and B0AT1 (*P* < 0.05) (Fig. 8a), and enhanced Gln transport by IEC-6 (*P* < 0.05) (Fig. 8b).

Compared with ITF treatment, after administration of 3-MA (5 mmol/L), the LC3-II/LC3-I ratio was substantially reduced (*P* < 0.05) (Fig. 8a), the protein levels of PDI, ASCT2, B0AT1 decreased enormously (*P* < 0.05) (Fig. 8a), the CHOP protein level increased sharply (*P* < 0.05) (Fig. 8a), and Gln transport by IEC-6 decreased (*P* < 0.05) (Fig. 8,b). The results of this study showed that after the suppression of autophagy, ERS was enhanced, the protein level of Gln transporters was reduced, and Gln transport by IEC-6 was impaired, which indicates that ITF indeed improves intestinal Gln transport by upregulating the autophagy level and promoting the protein levels of ASCT2 and B0AT1.

### Inhibition of AMPK phosphorylation mitigates the biological effect of ITF on IEC-6 cells

**Figure 9:**
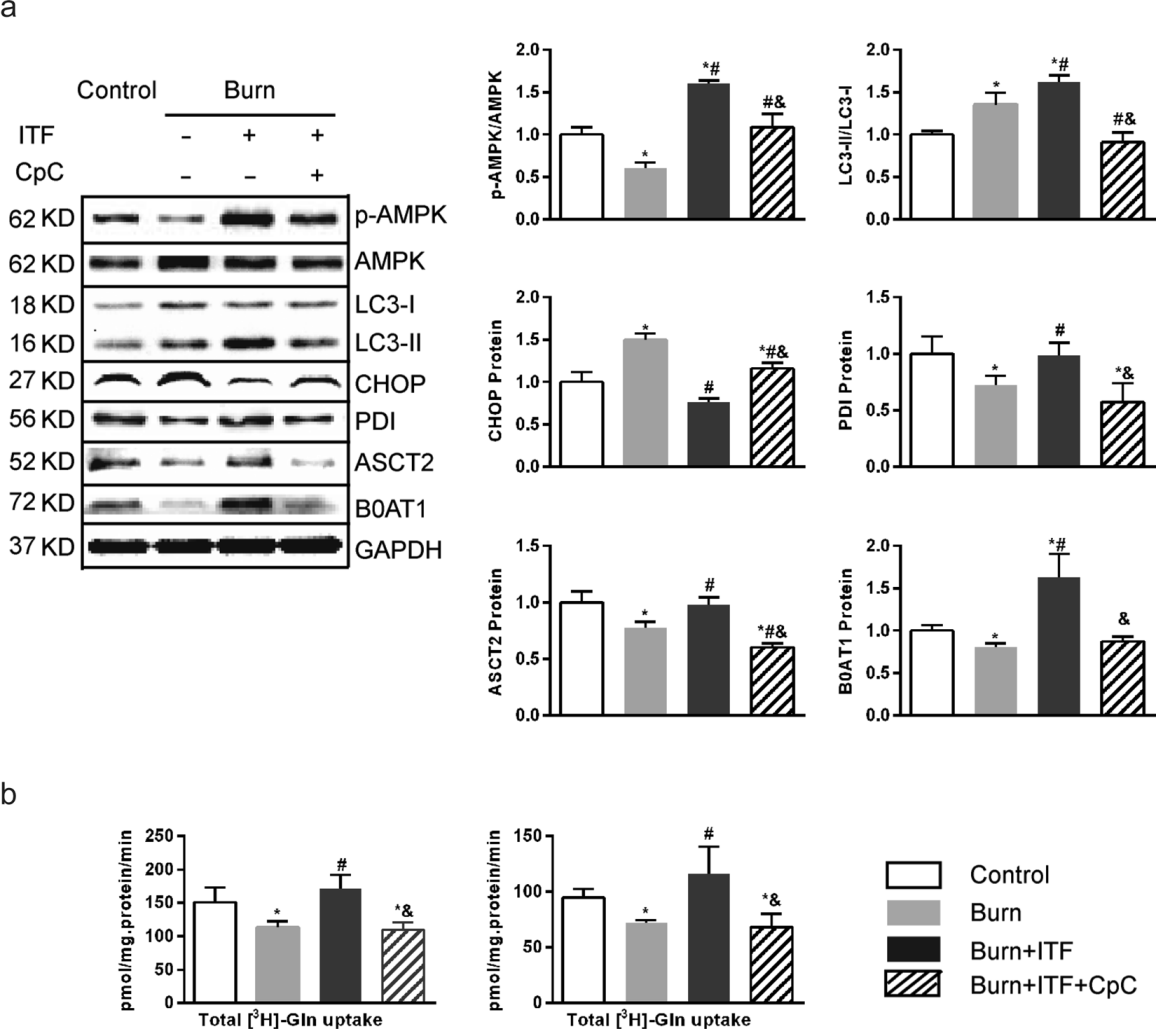
Inhibition of AMPK phosphorylation mitigates the biological effect of ITF on IEC-6 cells. (**a**) Western blotting analysis for protein levels of p-AMPM, LC3-II, CHOP, PDI, ASCT2, B0AT1. (**b**) Variation of glutamine transport in IEC-6 cells under different treatment factor. Datas were presented as means ± SD and analyzed by two-way ANOVAs. ^*^P < 0.05, compared to control; ^#^*P* < 0.05, compared to burned serum group; ^&^*P* < 0.05, compared to burned serum and ITF treated cells.

To confirm whether the AMPK phosphorylation is a key pathway by which ITF promotes the protein levels of ASCT2 and B0AT1 and improves Gln transport, this study applied compound C, an AMPK phosphorylation inhibitor^41^, to observe changes in the biological activity of ITF after AMPK phosphorylation suppression. The results showed that after IEC-6 cells were co-cultured with burn serum for 12 hours, the AMPK phosphorylation level decreased dramatically (*P* < 0.05) (Fig. 9a), the LC3-II/LC3-I ratio increased (*P* < 0.05) (Fig. 9a), the CHOP protein level increased markedly (*P* < 0.05) (Fig. 9a), the protein levels of PDI, ASCT2, and B0AT1 decreased significantly (*P* < 0.05) (Fig. 9a), while Gln transport by IEC-6 decreased substantially (*P* < 0.05) (Fig. 9b). Compared with the burn serum group, ITF administration significantly promoted AMPK phosphorylation level, increased the LC3-II/LC3-I ratio sharply (*P* < 0.05) (Fig. 9a), reduced the protein level of CHOP (*P* < 0.05) (Fig. 9a), promoted the protein levels of PDI, ASCT2, and B0AT1 (*P* < 0.05) (Fig. 9a), and enhanced Gln transport by IEC-6 (*P* < 0.05) (Fig. 9b).

Compared with ITF administration, after treatment with compound C (10 μmol/L) for 12 hours, the AMPK phosphorylation level was significantly inhibited (*P* < 0.05) (Fig. 9a), the LC3-II/LC3-I ratio was significantly reduced (*P* < 0.05) (Fig. 9a), the CHOP protein level increased substantially (*P* < 0.05) (Fig. 9a), the protein levels of PDI, ASCT2, and B0AT1 decreased significantly (*P* < 0.05) (Fig. 9a), and Gln transport by IEC-6 decreased (*P* < 0.05) (Fig. 9b). These results suggest that the inhibition of AMPK phosphorylation can mitigate the biological effect of ITF on IEC-6, as evidenced by weaker autophagy, enhanced ERS, suppressed protein levels of ASCT2 and B0AT1 and, ultimately, weaker Gln transport.

## Discussion

Glutamine in the intestinal lumen is transported to IECs mainly through microvilli that are neatly arranged in the brush border^42^. Chris IC *et al*. revealed microvilli curled into vesicle-like structures called BBMVs^43^. Under normal circumstances, BBMVs are shaped as spherical vesicles with a smooth surface and high refractivity. BBMVs that contain proteolytic enzymes, membrane receptors and various transporters function as a core part of the intestinal transport system^32^. The present study found that the structure of BBMVs was severely impaired after burn injury, as shown by such pathological changes such as varied shapes, rough borders, and vesicle collapse or even fractures. Structural damage further led to functional impairment of BBMVs in that Gln transport by BBMVs was decreased significantly. Because BBMVs account for the bulk of Gln transport in IECs, functional impairment of BBMVs may cause a decline in Gln transport in the entire intestine, which depends on sodium-dependent and independent Gln transporters in IECs. We revealed a sharp decline in sodium-dependent transporters but an increase in non-sodium-dependent transporters after burn injury. It is well known that sodium-dependent transporters are the dominant type of Gln transporters in IECs, especially in BBMVs. Consistently, we revealed that they account for about 75% and 85% of the total Gln transport in IECs and BBMVs, respectively (Fig. 1h,i).

Gln transport by IECs and BBMVs was decreased significantly after burn injury, which is associated with intestinal hypoxic-ischemic injury. Previous research has shown that intestinal blood perfusion decreases markedly and reaches a minimum 12 to 24 hours after injury, at a level approximately 20% of that before injury^44^. As time progresses after burn injury, the intestinal blood supply starts to improve, but it remains significantly lower on day 7 after burn injury, which is comparable with the change in the intestinal Gln transport after burn injury in our study. Tissue ischemia and hypoxia after burn injury disrupts energy metabolism and attenuates Na^+^-K^+^-ATP activity^45^ and sodium-dependent Gln transporter activity. In contrast, the enhanced activity of sodium-independent transporters may be a result of compensatory change. Because intestinal villi are more sensitive to ischemia and hypoxia, burn injury exerts a more notable influence on Gln transport by BBMVs, which leads to a direct decline in the ability of BBMVs to transport Gln and a lower percentage of Gln transported by BBMVs in the whole intestinal tract.

We showed that ITF significantly advanced Gln transport by IECs, especially by BBMVs in burned rats. Moreover, we found that ITF is more potent on the sodium-dependent transporters than on the non-sodium-dependent transporters as sodium-dependent Gln transporters dominate in BBMVs.

It was reported previously that ITF could lessen the stress responses triggered by intestinal damage, improve intestinal blood perfusion, and attenuate tissue damage after burn injury^46^. However, the underlying mechanisms are largely unclear in detail. We elucidate the main mechanism whereby ITF improves intestinal Gln transport after burn injury is that ITF alleviates hypoxic-ischemic damage to IECs and maintains the normal structure of IECs, especially BBMVs.

We investigated the effects of ITF on the protein levels of ASCT2 and B0AT1, two major subtypes of sodium-dependent Gln transporters. The present study found that the protein levels of ASCT2 and B0AT1 was significantly reduced after burn injury and ITF significantly rescued the protein expression of both transporters. However, our results showed that both mRNA levels of ASCT2 and B0AT1 in IECs kept nearly unchanged after burn, and ITF barely altered the mRNA levels of ASCT2 and B0AT1. These findings suggest ITF increases ASCT2 and B0AT1 protein levels likely by promoting their translation and regulating post-translational modification.

ASCT2 and B0AT1 are cysteine-containing transporter proteins with special space structures^18^. Upon peptide chain synthesis, the cysteine residues in the two transporters are catalyzed by PDI to generate disulfide bonds in the ER to form a correct spatial structure. Thus, normal function of the ER has a direct bearing on the modification of these transporter proteins. Our study reveals that severe burn-induced ERS in IECs because the expression of CHOP and GRP-78, two ERS markers were increased but the expression and activity of PDI were reduced. Previous studies reported the disturbance of redox equilibrium in the process of ERS. PDI, a chaperone in the ER, sustains oxidative damage and inactivation, resulting in unfolded or misfolded proteins due to the failure to form disulfide bonds after peptide chain synthesis and subsequent accumulation of abnormal proteins^33,47^. Consistently, we found the expression and activity of PDI were decreased during the severe burn-induced ERS in IECs, ultimately leading to the failure of post-translational modification on ASCT2 and B0AT1.

With a decrease in PDI activity after burn injury, large amounts of proteins in the ER cannot be properly modified, resulting in ERS^48,49^. Marie PC *et al*.^31^ reported that TFF1, a member of the same protein family as ITF, maintained the structure and function of the ER. We demonstrate that ITF significantly lessened ERS since it decreased the expression of CHOP and GRP-78, but increased the expression and activity of PDI, which is consistent with the changes in ASCT2 and B0AT1 and sodium-dependent Gln transport. To further test if ITF promotes Gln transport through PDI, we applied 16F16 to inhibit the PDI activity and found that the effects of ITF were significantly suppressed, indicating ITF indeed promotes Gln transport through activating PDI.

Autophagy is a catabolic process for the degradation of redundant or damaged cell components and alleviates ERS by removing unfolded or misfolded proteins. ERS and autophagy interact and jointly maintain cell homeostasis^39,40,50^. The present study found that autophagy was enhanced in IECs after burn injury, which may protect IECs in response to stress. However, enhanced autophagy is insufficient to clear the accumulation of large numbers of abnormal proteins caused by burn injury, and ERS remains serious. In the present study, ITF significantly enhanced autophagy in IECs, while at the same time, ERS was decreased to the minimum, suggesting that the enhanced autophagy inhibits ERS. The inhibition of autophagy using 3-methyl adenine (3-MA) significantly suppressed the effect of ITF on the enhanced autophagy. Furthermore, 3-MA significantly increased ERS and reduced protein levels of PDI, ASCT2, and B0AT1, ultimately leading to a sharp decline in the ability of ITF to promote intestinal Gln transport. Therefore, we showed that autophagy is a key step to regulate ERS in IECs and is closely related to the synthesis and activity of Gln transporters.

It is well known that AMPK induces autophagy by reducing the activity of the mammalian target of rapamycin (mTOR)^35–37^. The present study showed that AMPK phosphorylation was reduced in rat IECs after burn injury but ITF improved post-burn AMPK phosphorylation. ITF induced the parallel changes in the levels of AMPK phosphorylation and autophagy in IECs, suggesting that it can enhance autophagy via AMPK pathway. Activated AMPK can not only alleviate the damage by abnormal proteins in the ER by activating autophagy but also reduce cell damage by maintaining energy metabolism homeostasis and ameliorating oxidative stress. T. Yano *et al*. demonstrated that AMPK activation plays an important role in maintaining the structure of IECs and the integrity of the intestinal mucosal barrier^51^. The present study showed that ITF activates AMPK, providing an insight into the mechanism by which ITF enhances autophagy and reduces post-burn ERS to maintain intestinal mucosal barrier by improving oxidative stress and enhancing epithelial tight junctions. In summary, we showed that ITF can significantly enhance intestinal Gln transport in burned rats and improve the utilization of enteral Gln supplementation. Mechanistically, ITF enhances autophagy, reduces ERS, and alleviates the impaired PDI, ASCT2, and B0AT1 in IECs after burn injury likely through AMPK activation. Therefore, ITF may be therapeutically useful to improve intestinal glutamine transport by alleviating burn-induced ERS.

## Materials and Methods

### Animal handling

Adult male Sprague-dawley rats were purchased from Third military medical university animal experiment center and allowed to acclimate for one week prior to the experiment. Rats were housed in a temperature-controlled cubicle with a 12-hour light/dark cycle with laboratory chow and water ad libitum. All animal procedures were performed in adherence to the southwest hospital for the Care and Use of Laboratory Animals, according to the protocol outlined in the Guide for the Care and Use of Laboratory Animals published by the US National Institute of Health (NIH publication no. 85–23, revised 1996), and the experimental protocols were performed in accordance with the approved guidelines.

A well-established method with minor modifications was applied to induce a full-thickness scald burn^52^. 120 Sprague-dawley rats were randomly divided into three groups, namely normal group (n = 40), burned group (n = 40) and burned + ITF group (n = 40). Each group included four time points that are 1 day, 3days, 5days and 7days post burn or ITF administration. Every observation phase include 10 rats in per group. All rats were anesthetized with 1% pentobarbital sodium (40 mg/kg body weight i.p) and buprenorphine (0.05 mg/kg body weight) was administered for analgesia. The dorsal surfaces were shaved, rats were placed to a mold with an opening providing a 30% total body surface area burn. The rats in control group were immersed in 37 °C water. Rats from other groups were then immersed in 97 °C water for 18 seconds on the dorsal. Lactated Ringers solution (40 ml/kg body weight i.p.) was then administered intraperitoneally for resuscitation. Rats were housed in separate cages. In group burned + ITF, rats were accepted gavage ITF (1 mg/kg body weight), bid, rats in other groups were perfused with the same volume of saline. All rats were sacrificed at 1 day, 3days, 5days, 7days after burn. Small intestine was excised immediately for preparing Brush BBMVs and IECs.

### IECs preparation

IECs were isolated from the small intestine by a calcium chelation technique^53^. Briefly, a 60 cm section of small intestine was separated and the half of the section was rinsed thoroughly with D-Hanks water, then it was cut into tissue pieces and collected in a culture bottle containing 20 mL cell isolation buffer (0.15 mM EDTA, 112 mM NaCl, 25 mM NaHCO_3_, 2.4 mM K_2_HPO_4_, 0.4 mM KH_2_PO_4_, 2.5 mM L-glutamine, and 0.5 mM dithiothreitol, pH 7.4) for 30 minutes and gently palpitated to facilitate cell dispersion at 37 °C. The buffer was then collected, phenylmethylsulfonyl fluoride was added, and the suspension was centrifuged at 100 g for 5 minutes. Cells were been used for uptake studies of [^3^H]-Gln and extraction of total protein (4–5 mg/ml).

### BBMVs preparation

A half intestine section was flushed gently with 30 ml of ice-cold isotonic saline containing 0.1 mM PMSF and placed on an ice-cold plate. The intestine was opened along its length, and the tissue was laid flat. The mucosa was scraped off using an ice-cold glass slide and shaken in a homogenizer that contains 8 ml of collection solution A (300 mM mannitol, 5 mM EGTA, 12 mM Tris·Cl, pH 7.4). The mucosa suspension was homogenized for 2 minutes and then for 5 minutes on ice. Then, 2 ml of ice-cold 50 mM MgCl_2_ as added to the homogenate. This solution was kept on ice for 15 minutes and gently stirred continuously using a magnetic stirrer^43^. Finally, the protein concentration of the BBMVs was determined using standard protocols. BBMVs were used for uptake studies of [^3^H]-Gln and transmission electron experiments^43^.

### Cell culture

Rat small intestinal epithelium cell line (IEC-6) was purchased from American Type Culture Collection (ATCC Cell Biology, Catalog No. CRL-1592). Cells were grown in a humidified incubator at 37 °C in 5% CO_2_/94% O_2_/1% N_2_ and were routinely grown in high-glucose Dulbecco’s modified Eagle medium (DMEM) that contained 10% fetal bovine serum (FBS), 4 mmol/L glutamine, 100 IU/mL penicillin, and 100 μg/mL streptomycin. IEC-6 cells were dissociated with 0.05% trypsin and 0.02% ethylenediamine-tetraacetic acid (EDTA) when cell confluence was over 80%. Cells were seeded in 6-well cluster Costar tissue culture plates at a density of 100,000 cells per well for Western blot analysis and seeded in the 24-well cluster Costar tissue culture plates at a density of 10,000 cells per well for the [^3^H]-Gln transport experiments. When the cell confluence was over 60%, the IEC-6 monolayer was then washed 2 times with PBS, and the culture medium was replaced by DMEM high glucose medium that contained 10% rat serum or burn rat serum (burn serum was collected and isolated from rats exposed to burns for 3 days), or burn rat serum and 10 μg/mL ITF, or burn rat serum and 10 μg/mL ITF and compound C/3-MA/16F16, with 4 mmol/L glutamine. For 12 hours, cell lysate was collected, and protein was extracted for Western blot analysis. Cells in the 24-well cluster Costar tissue culture plates were used for the [^3^H]-Gln transport experiments.

### Uptake studies of [^3^H]-Gln

Uptake buffer (37 °C) was composed of 137 mM NaCl (or 137 mM choline chloride), 4.7 mM KCl, 1.2 mM MgCl_2_, 1.2 mM KH_2_PO_4_, 2.5 mM CaCl_2_, and 10 mM HEPES/Tris buffer (pH 7.4). Uptake studies in 100 μL rat IECs or 40 μL BBMVs suspension (4–5 mg/ml) were initiated by simultaneously adding 1 mL of this buffer, which also contained [^3^H]-L-glutamine (0.5 μCi/mL, 20 μmol/L), into each transport plate for 1 minute; the rat IECs or BBMVs were washed and filtered on filter paper, and then the radio-activity was assayed by liquid scintillation spectrometry. IEC-6 cells in 24-well cluster Costar tissue culture plates were rinsed 2 times with uptake buffer. Transport was initiated by simultaneously adding 1 mL of this buffer, which also contained [^3^H]-L-glutamine (0.5 μCi/mL, 20  μmol/L) into each of 24 wells in a transport plate. Cell culture plates were continuously shaken by an orbital shaker (1 Hz) during the transport period. After 1 minute, the assay was terminated by discarding the uptake buffer and rinsing the cells 3 times with ice-cold uptake buffer. The cells were solubilized by 1 mL of 0.2-N NaOH/0.2% SDS solution. The radio-activity of the isotope extracted from the cells was assayed by liquid scintillation spectrometry. Uptake rates are expressed as pmol of [^3^H]-glutamine per minute per mg of cells or BBMVs protein. Sodium-dependent glutamine transport values were obtained by subtracting the total glutamine transport measured in choline chloride buffer from that in NaCl buffer^54^.

### Western Blotting

Protein level of ASCT2 (1:1000 Sigma), B0AT1 (1:200 Santa), p-AMPK (1:1000, Abcam, UK), AMPK (1:1000, Abcam, UK), CHOP (1:1000 CST), GRP-78 (1:1000 CST), PDI (1:1000 CST), SNO-PDI (1:1000 CST), LC3-II (1:1000 Sigma), p62 (1:1000 CST) in IECs from SD rats or IEC-6 cells that underwent different treatments were analyzed by Western blotting using commercially available antibodies. The cell lysates were subjected to sodium dodecyl sulfate (SDS) (Amresco, OH, USA)-PAGE and subsequently transferred to a polyvinylidene difluorid (PVDF) membrane (Millipore Immobilon, USA). Following washes with TBST, protein bands were detected using enhanced chemoluminescence ECL (Healthcare, Buckinghamshire, United Kingdom) according to the manufacturer’s instructions.

### Morphologic observation for BBMVs

A copper network with a membrane was placed into homogeneous BBMV suspension and rested for 10 minutes, and then excess liquid around the copper network was absorbed with filter paper, following dropping negative staining water on the network (potassium phosphotungstate), waiting for 10 seconds, quick drying and observing by transmission electron microscope (Third military medical university center lab).

### Q-PCR

Total RNA was extracted and purified from the intestinal mucosa samples with the TRIzol Reagent protocol (Invitrogen, Carlsbad, CA, USA). Total RNA was quantified using the BECKMAN COULTER DU800 nucleic acid/protein analyzer. The first-strand cDNA was synthesized by reverse transcription from 2 μg of total RNA with First Strand cDNA Synthesis Kit (TOYOBO, OSAKA JAPAN). The primers sets for GAPDH were 5′-GAA GGG CTC ATG ACC ACA GT-3′ (forward) and 5′-GGA TGC AGG GAT GAT GTT CT-3′ (reverse). The primers sets for ASCT2 were 5′-CCT AGA CCT GGG ATC ACG GA-3′ (forward) and 5′-CAG ATC AGG ACG TAG CGG TC-3′ (reverse). The primers sets for B0AT1 were 5′-CAT GTT CGT GTC CTT CAT GG-3′ (forward) and 5′-GTT CTG GTT GAG TGG GCA TT-3′ (reverse). The primers sets for GRP-78 were 5′-CAAGAACCAACTCACGTCCA-3′ (forward) and 5′-ACC ACC TTG AAT GGC AAG AA-3′ (reverse). The primers sets for CHOP were 5′-CTG GAA GCC TGG TAT GAG GA-3′ (forward) and 5′-GGG ATG CAG GGT CAA GAG TA-3′ (reverse). The primers sets for PDI were 5′-CTG CCC AAG AGT GTG TCT GA-3′ (forward) and 5′-TAT GCG CTG GTT GTC AGT GT-3′ (reverse). They were designed and synthesized by Sangon Biotech (CN). The Q-PCR was performed using the SYBR Green Realtime PCR Master Mix (TOYOBO, OSAKA JAPAN) on an Applied Biosystems 7500 Real-Time PCR System (Foster City, CA, USA). The delta cycle threshold (*C*T) method was used to analyze the relative expression of the target gene.

### PDI activity assay

The PDI activity was detected with insulin degradation, as described previously. Briefly, rat intestinal mucosa samples lysates were mixed with buffer (1 mM DTT, 30 μM insulin, and 3.0 mM sodium EDTA, in a buffer that contained 100 mM sodium phosphate). The precipitation of the insulin B chain was measured by an increase in absorbance at 610 nm.

### PDI S-Nitrosylation assay

SNO-PDI was tested using the biotin-switch assay, as described previously. Briefly, protein samples were added to a mix with 20 mM methylmethanethiosulfonate and 2.5% SDS in HEN buffer and were incubated at 50 °C for 1 h. Acetone was used to remove methylmethanethiosulfonate, and then, we used 1 mM ascorbate to reduce the free thiols. The formed thiols were linked to the biotin-HPDP. The biotinylated proteins were pulled down, and the SNO-PDI that remained in the precipitation was detected by western blotting.

### Statistical analysis

All datas were expressed as means ± SD. Since our experimental design contain two variates. One being the treatment factor and the other the time factor, two-way analyses of variance (Two-Way ANOVAs) was performed on the datas. All statistical analysis were performed using SPSS 13.0. *P* < 0.05 was considered statistically significant.
