# Author Correction: Role of intestinal trefoil factor in protecting intestinal epithelial cells from burn-induced injury

**DOI:** 10.1038/s41598-020-69648-x

**Published:** 2020-07-24

**Authors:** Jianhong Hu, Yan Shi, Chao Wang, Hanxing Wan, Dan Wu, Hongyu Wang, Xi Peng

**Affiliations:** Institute of Burn Research, State Key Laboratory of Trauma, Burns and Combined Injury, Southwest Hospital, The Third Military Medical University, Chongqing, 400038 China

Correction to: Scientific Reports 10.1038/s41598-018-21282-4, published online 16 February 2018

This Article contains errors.

As a result of errors during the figure assembly, in Figure 2 different frames from the same original image for “Burn + ITF/7 days” sample were used also for the image of the “Control/1 day” sample and the image for “Burn + ITF/5 days” sample. An incorrect image was also used for “Control/3 day” inset. The correct Figure 2 appears below as Figure 1.

**Figure 1:**
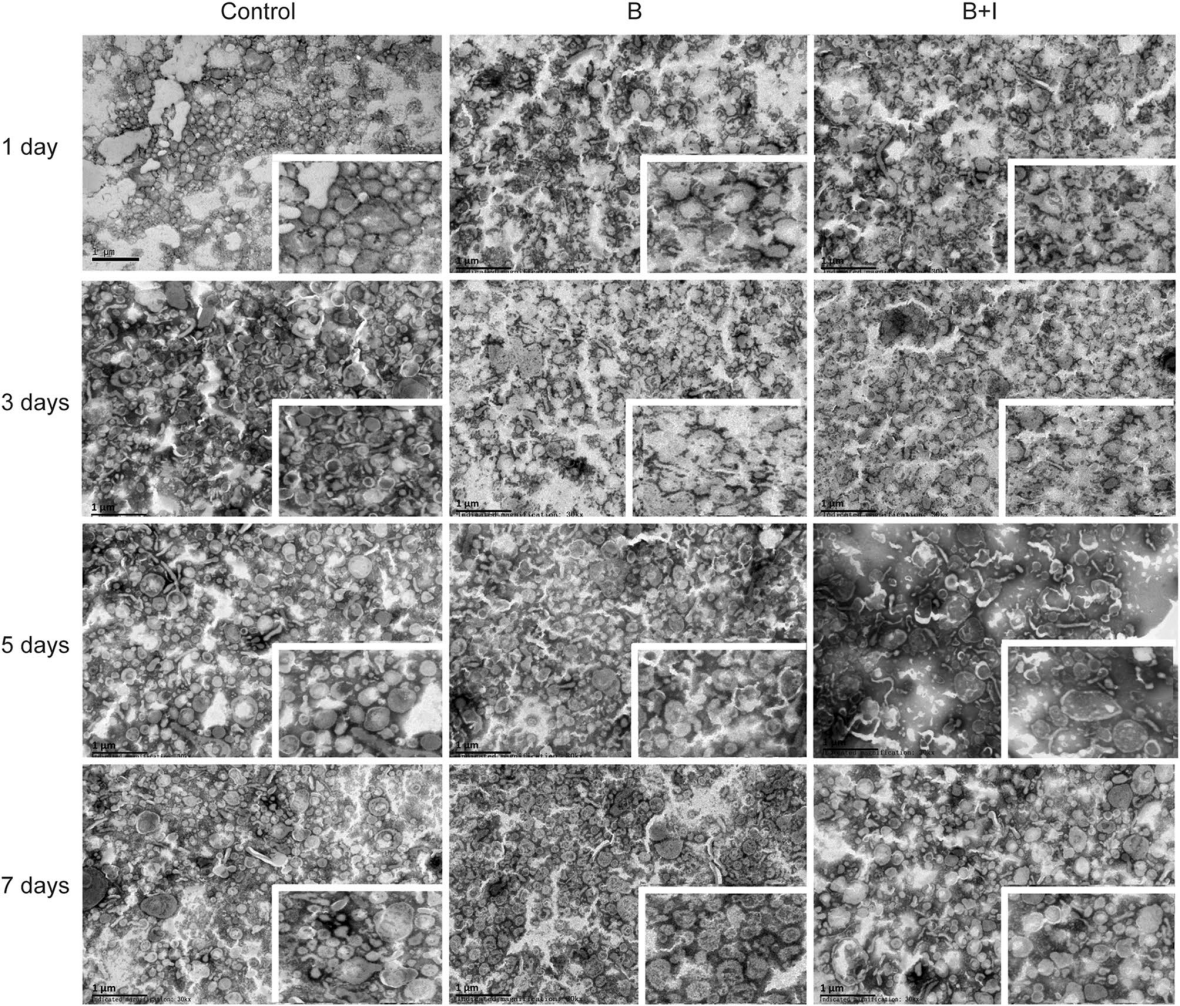
Effect of ITF on the burn-induced morphological change in BBMVs of IECs. The morphological structure of BBMV was observed by transmission electron microscope (bar, 1 μm) (n = 10/group per time point).

Additionally, a Supplementary Information file containing original data for Figure 2 as well as for all Western blots shown in the Article is now included with this correction notice.

## Supplementary information

Supplementary Information.

