# Supplementary material for: Author Correction: Role of intestinal trefoil factor in protecting intestinal epithelial cells from burn-induced injury
Source: Sci Rep. 2020 Jul 24;10:12741. doi: 10.1038/s41598-020-69648-x (PMC7378820; doi:10.1038/s41598-020-69648-x)

# Role of intestinal trefoil factor in protecting intestinal epithelial cells from burn-induced injury

Jianhong Hu, Yan Shi, Chao Wang, Hanxing Wan, Dan Wu, Hongyu Wang & Xi Peng

**Figure 2.** Effect of ITF on the burn-induced morphological change in BBMVs of IECs

Control 1 day

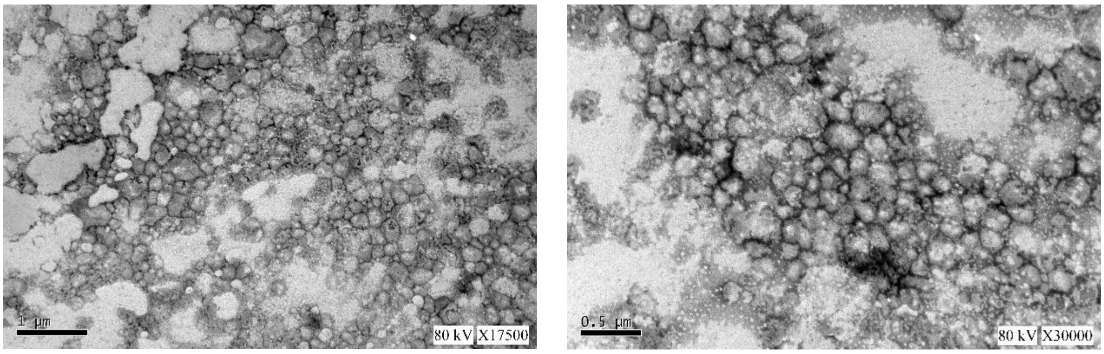


Burn 1 day

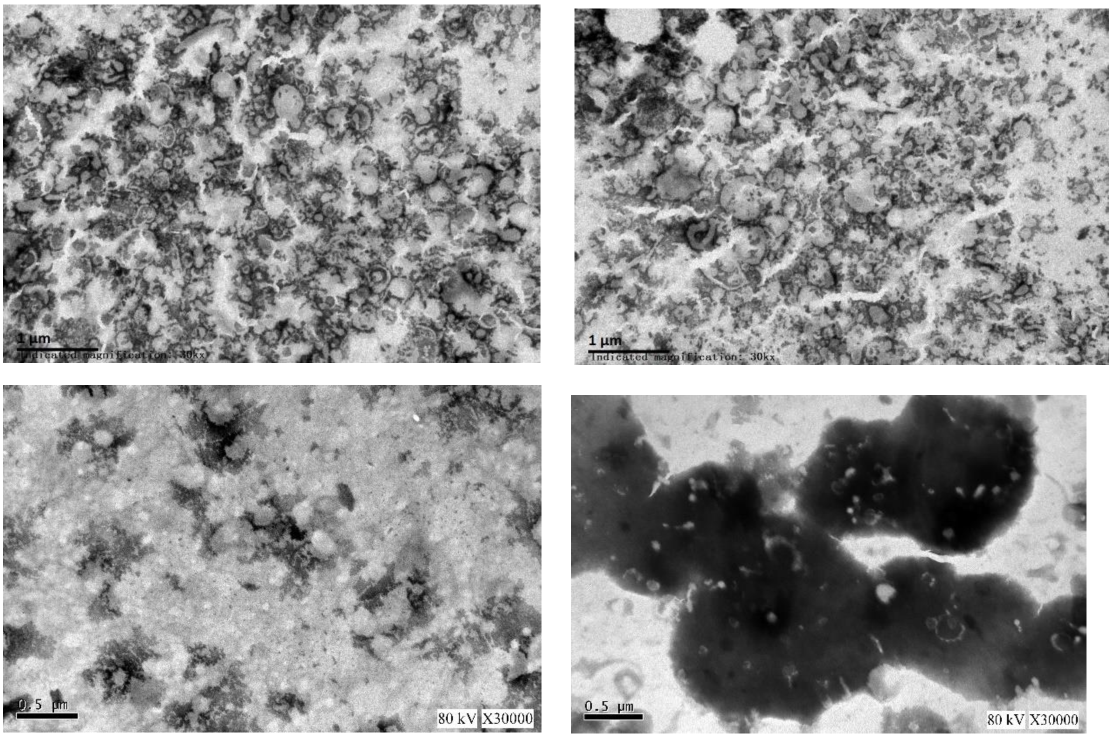


Burn +ITF 1 day

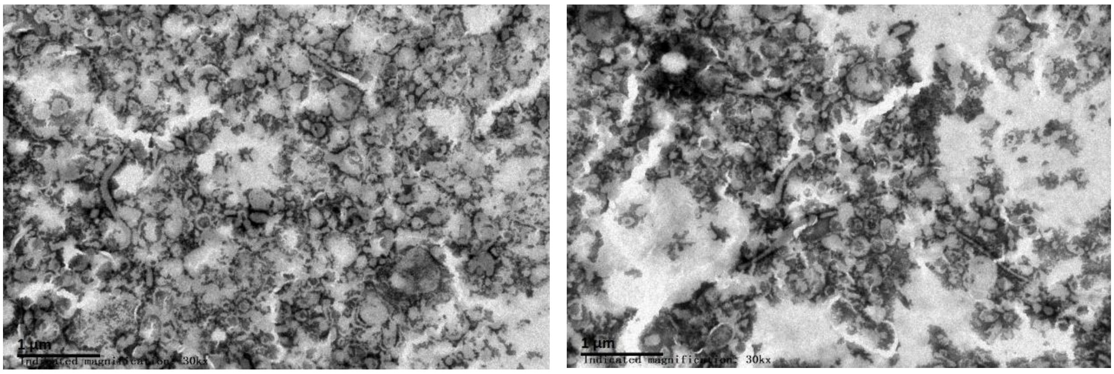

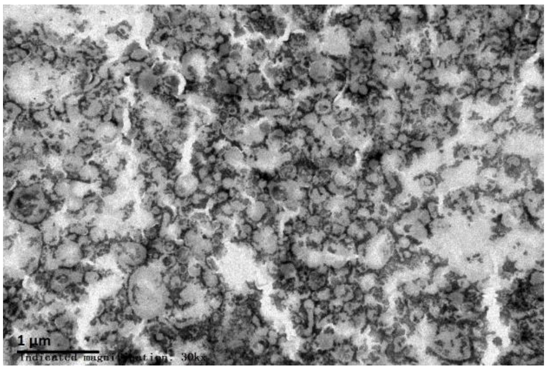


Control 3 day

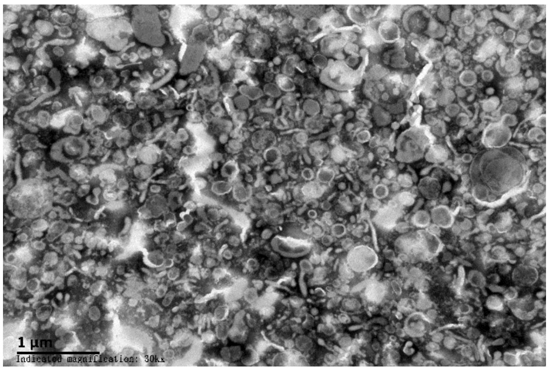


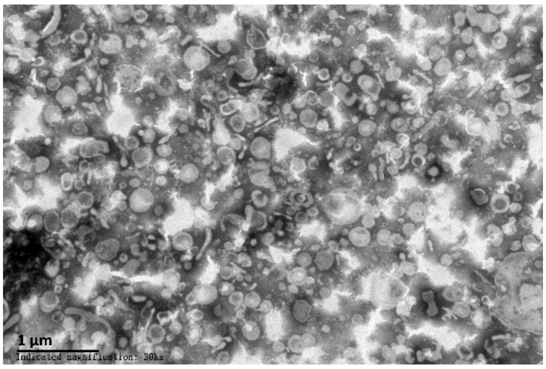


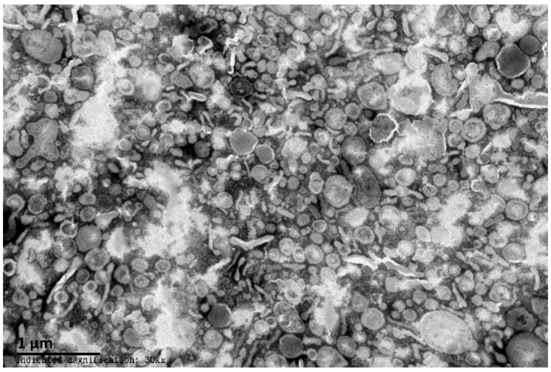


Burn 3 day

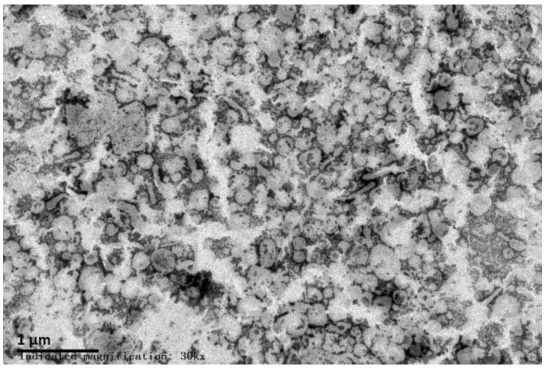


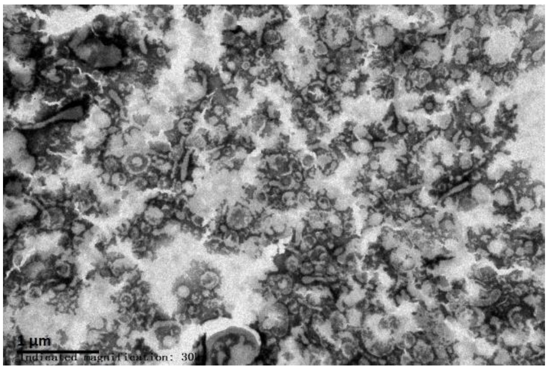

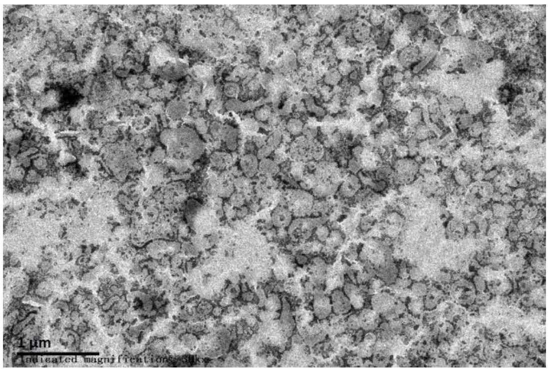


Burn +ITF 3 day

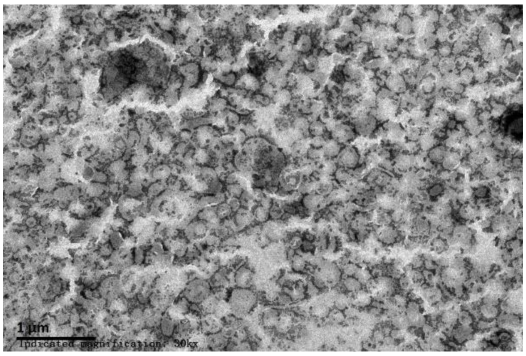


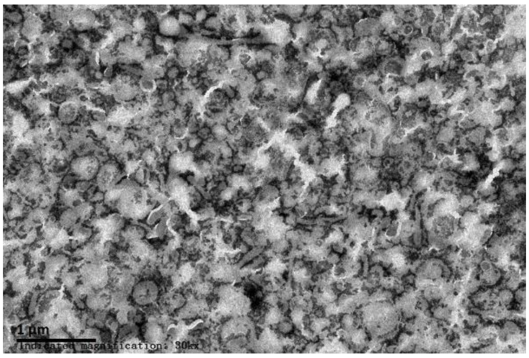


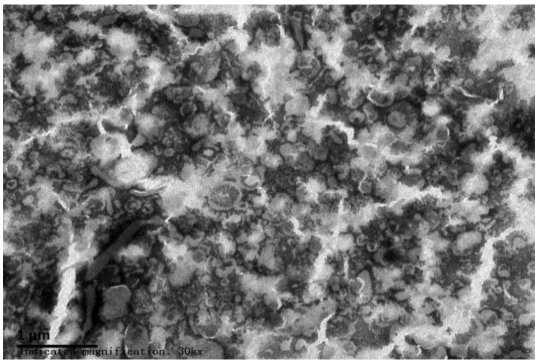


Control 5 day

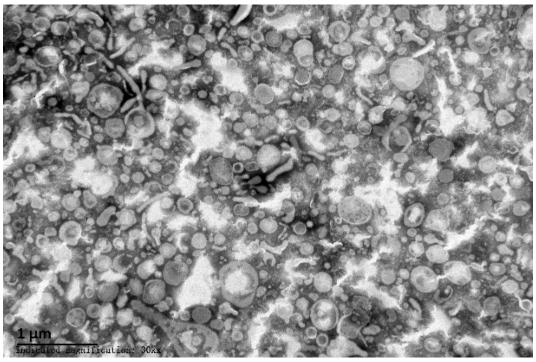


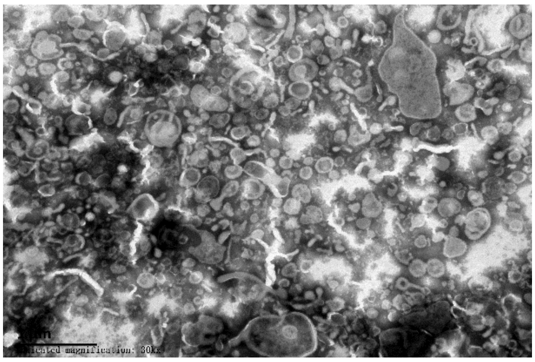

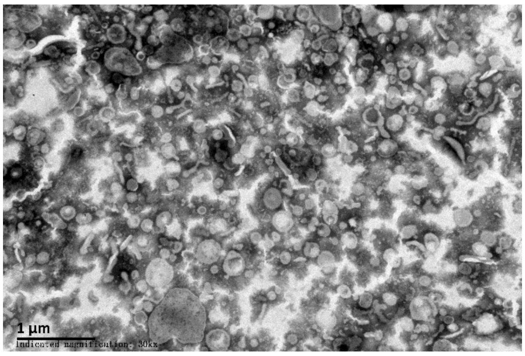
1 µm
Indicated magnification: 30kx

Burn 5 day

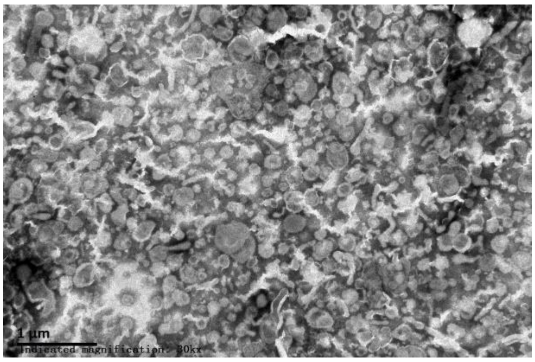
1 µm
Indicated magnification: 30kx

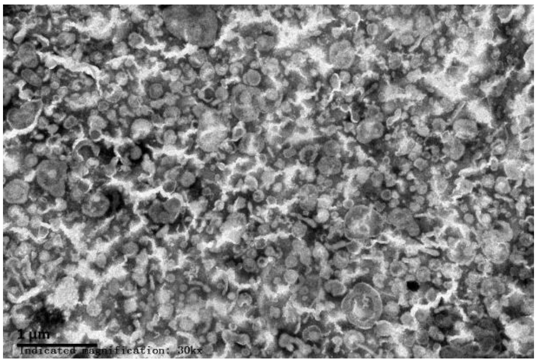
1 µm
Indicated magnification: 30kx

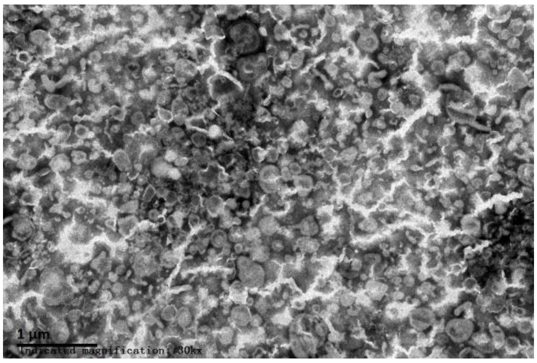
1 µm
Indicated magnification: 30kx

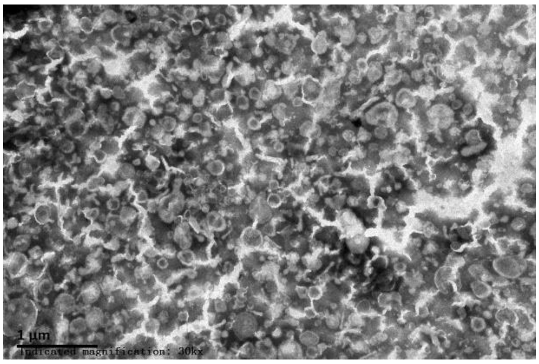
1 µm
Indicated magnification: 30kx

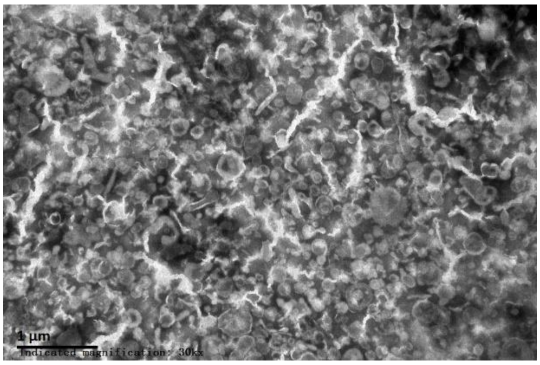
1 µm
Indicated magnification: 30kx

Burn +ITF 5 day

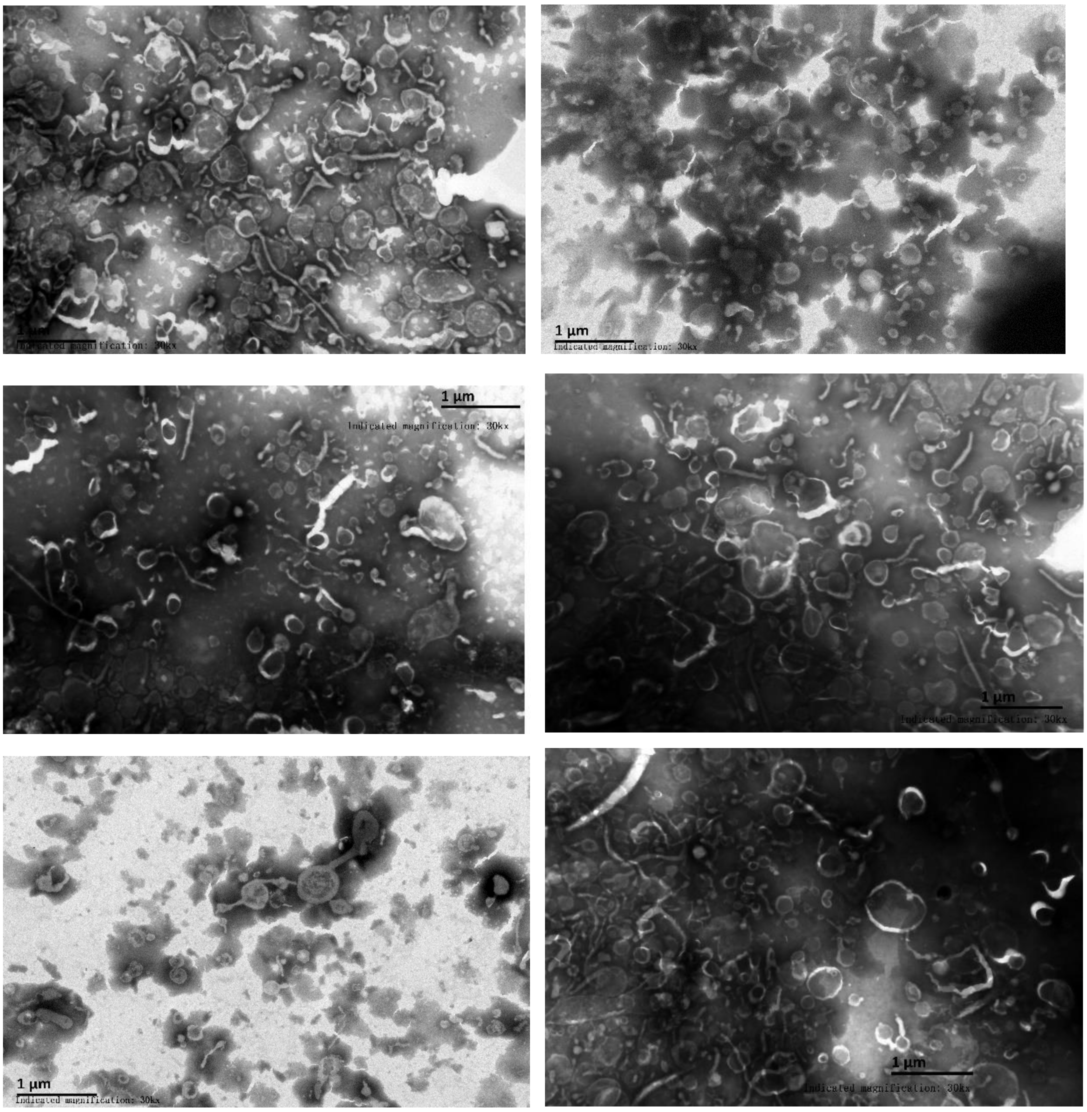


Control 7 day

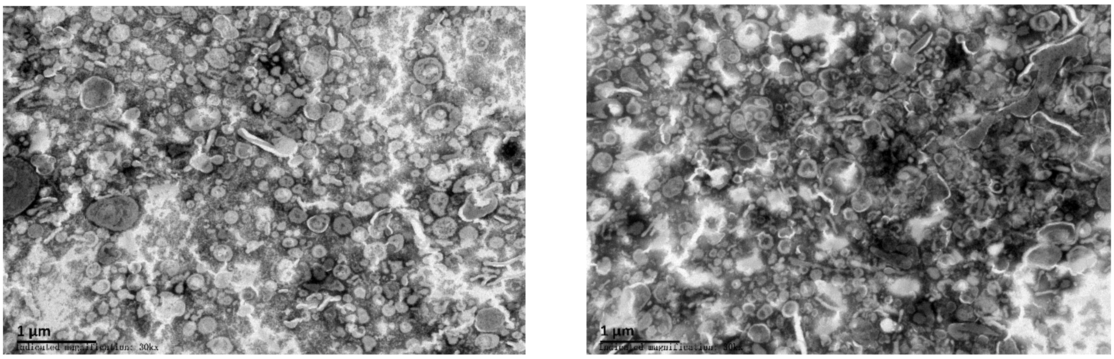

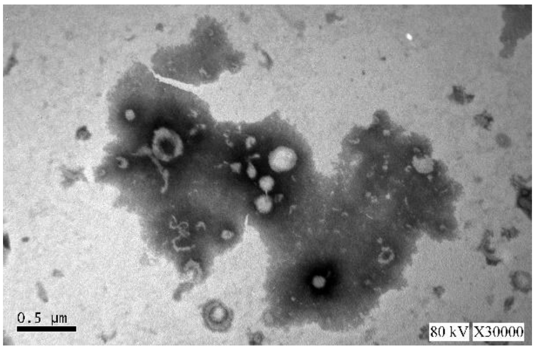


Burn 7 day

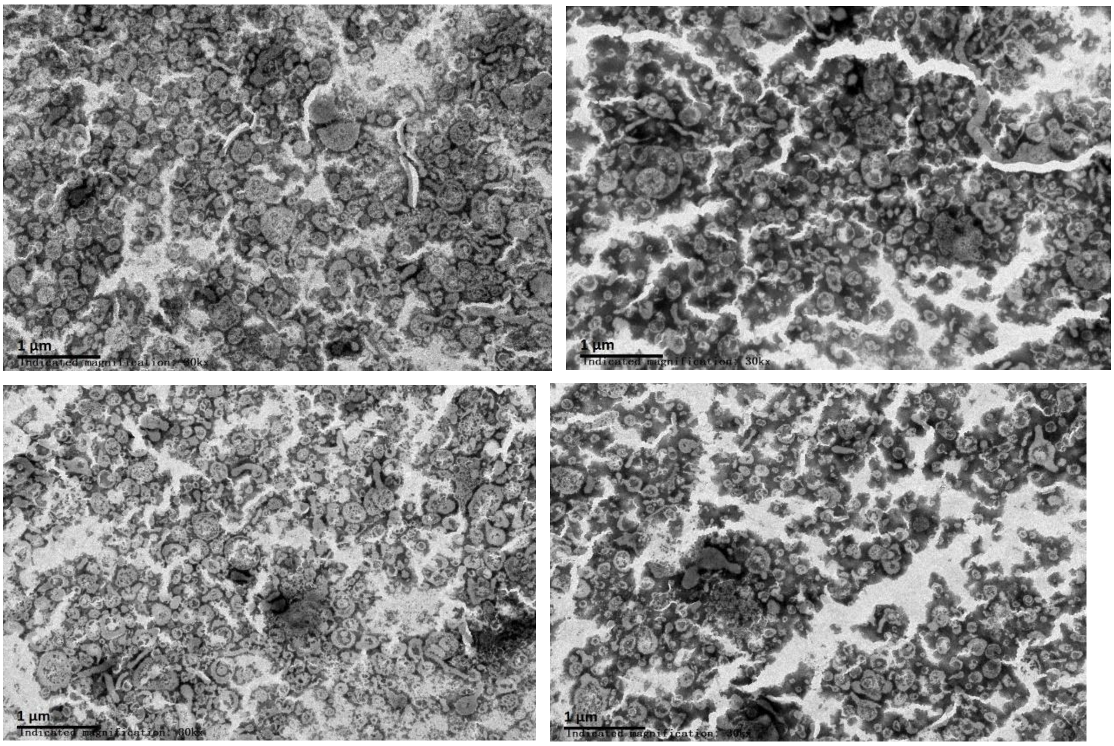


Burn +ITF 7 day

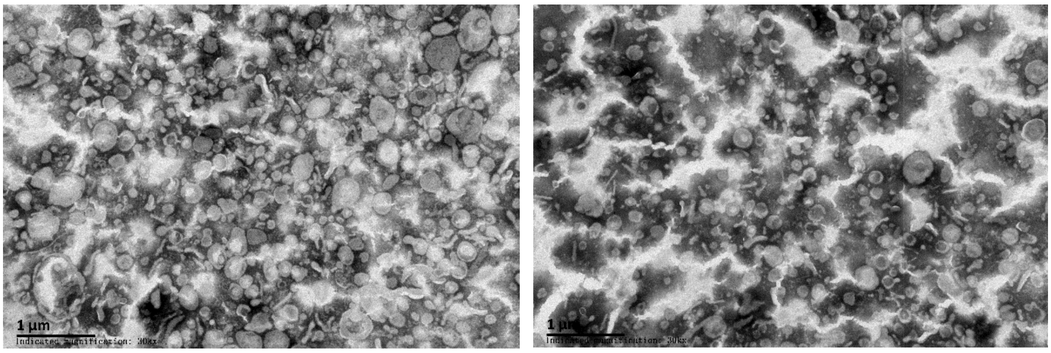

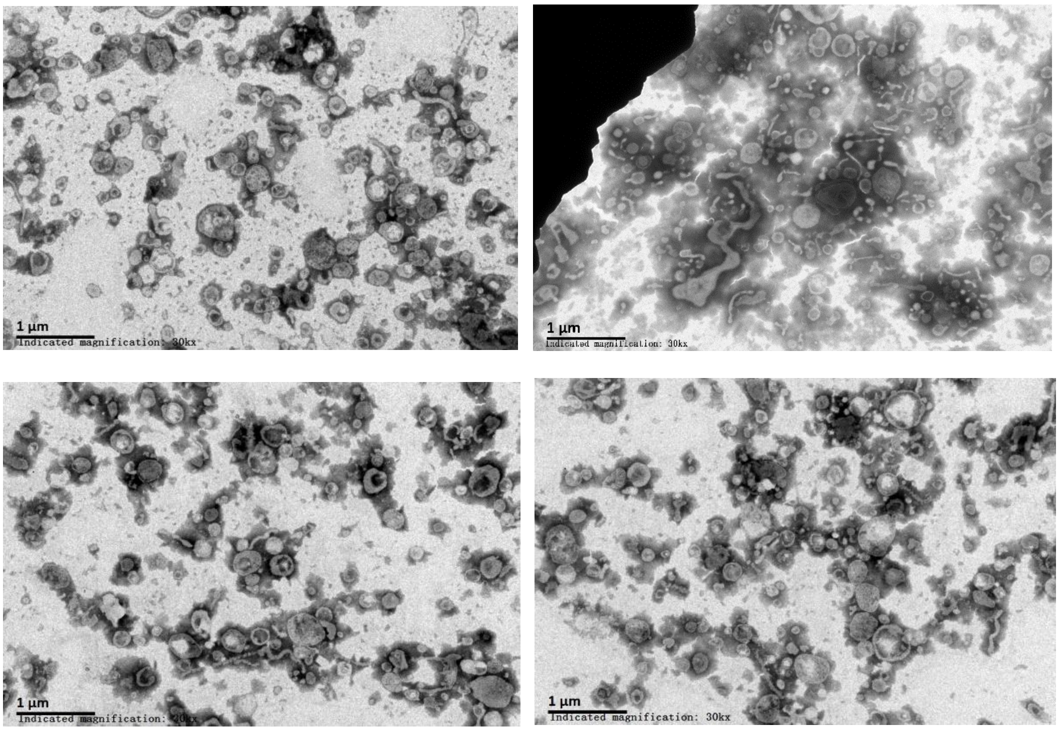


**Figure 3.** Effect of ITF on the burn-induced changes in the expression of ASCT2 and B0AT1 in IECs by western bloting.

1 day

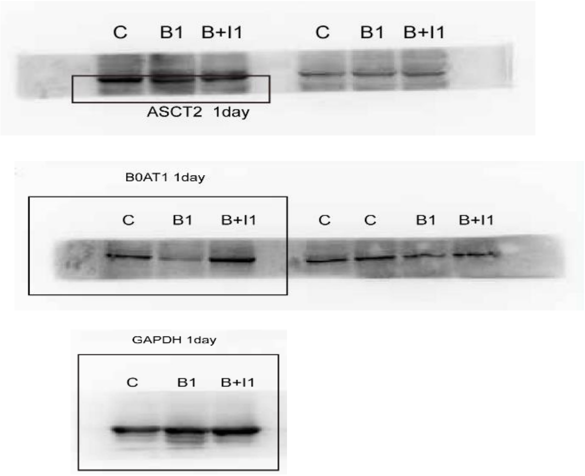

3day

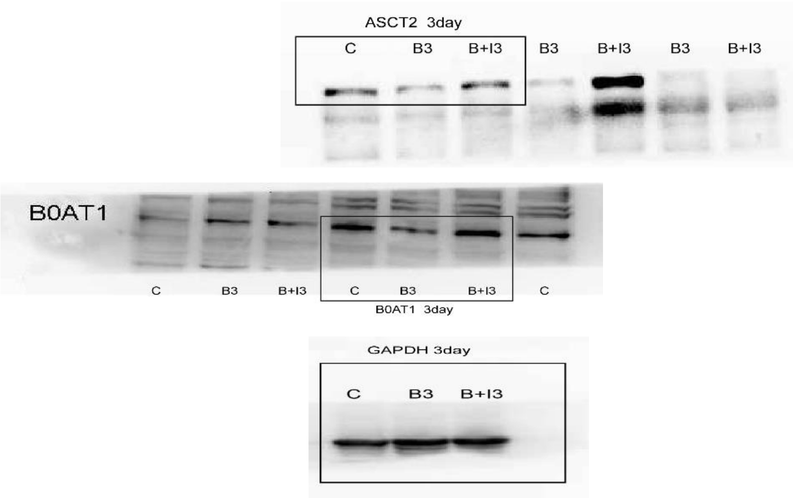


5day

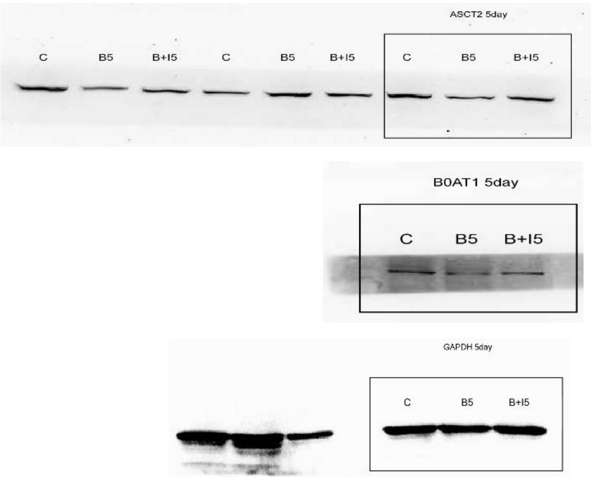


7day

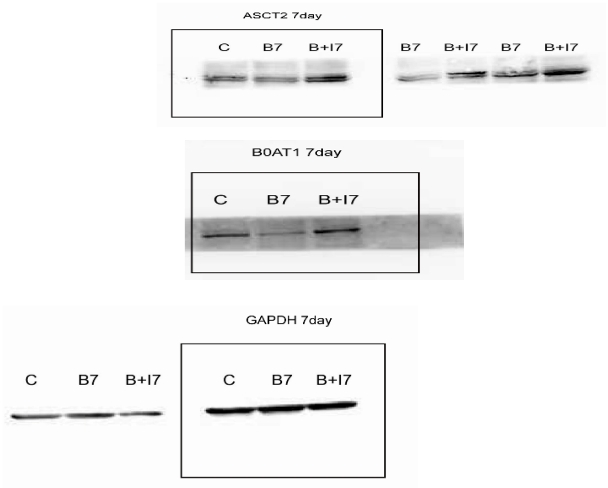

**Figure 4.** Effect of ITF on the burn-induced change in ERS indicators of IECs.

1day

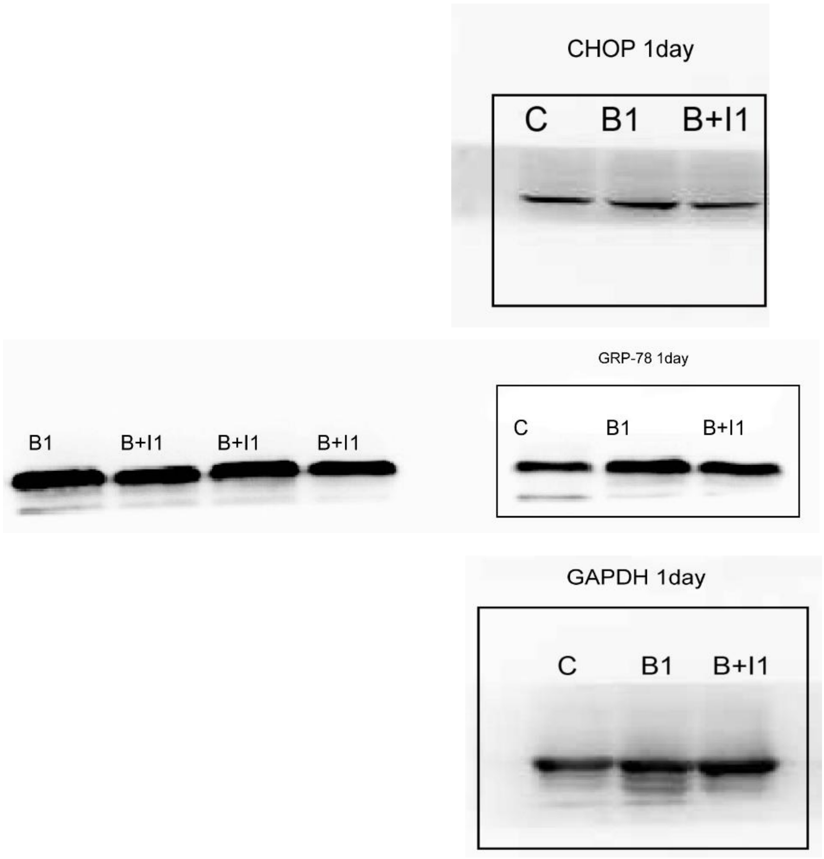


3day

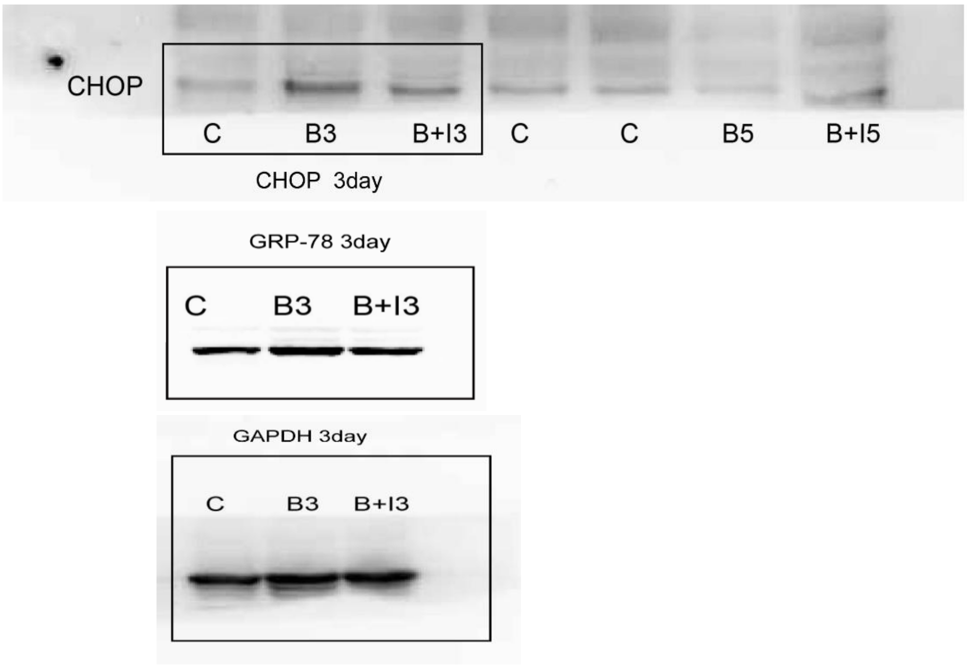

5day

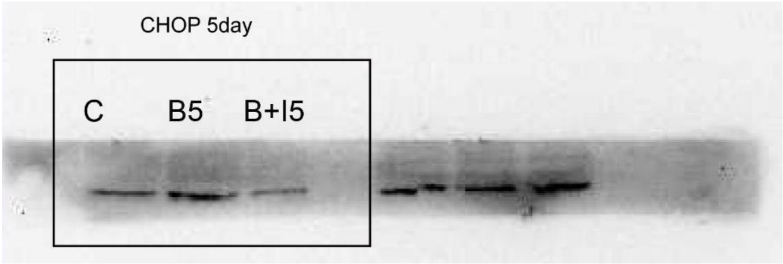
CHOP 5day
C
B5
B+I5

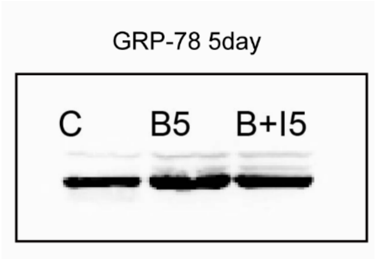
GRP-78 5day
C
B5
B+I5

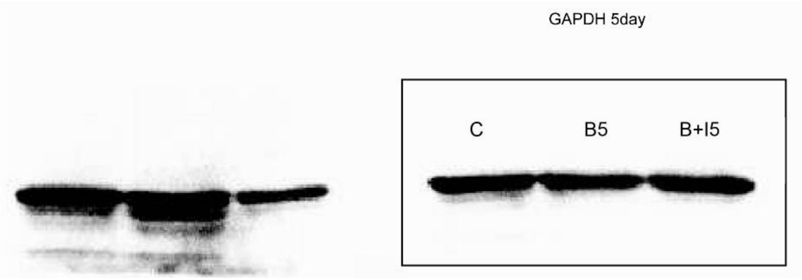
GAPDH 5day
C
B5
B+I5

7day

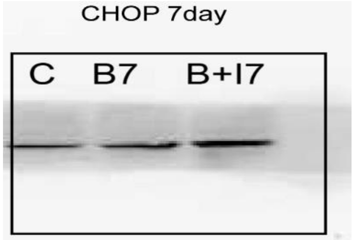
CHOP 7day
C
B7
B+I7

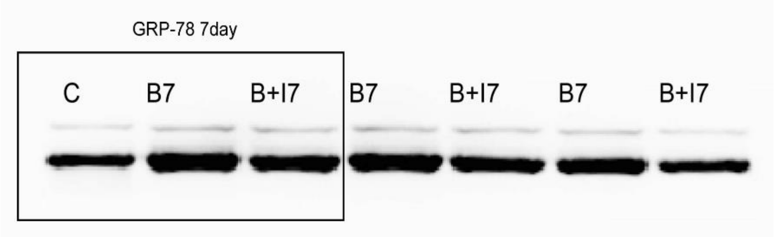
GRP-78 7day
C
B7
B+I7
B7
B+I7
B7
B+I7

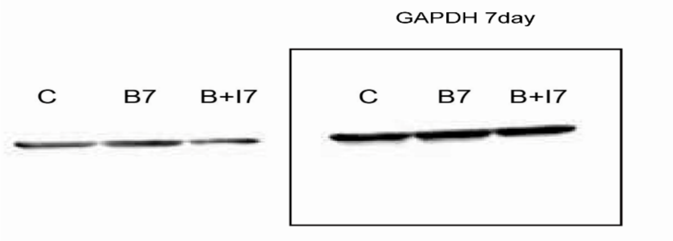
GAPDH 7day
C
B7
B+I7
C
B7
B+I7

**Figure 5.** Effect of ITF on the burn-induced change in protein disulfide isomerase in IECs.

1day

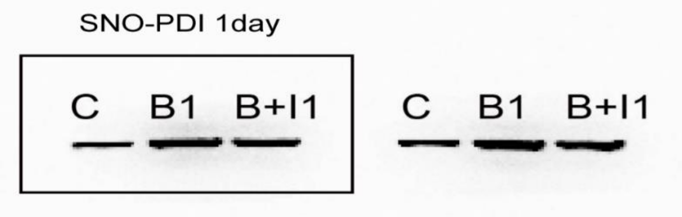


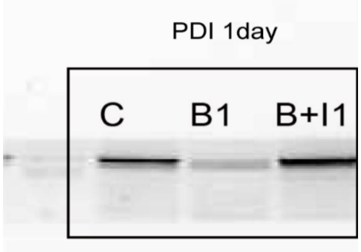


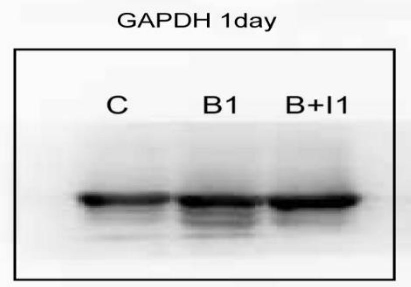


3day

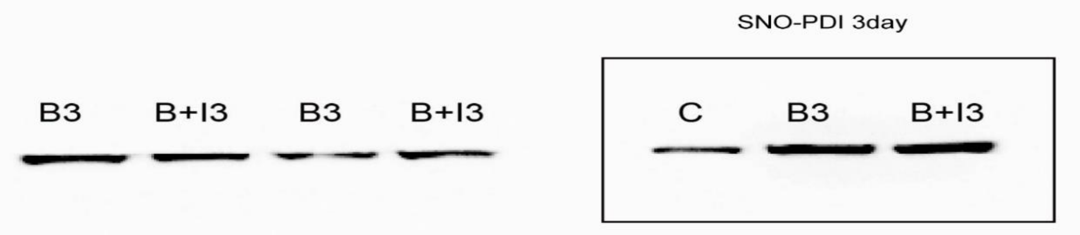


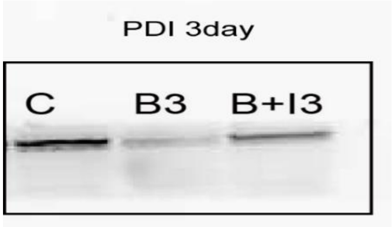


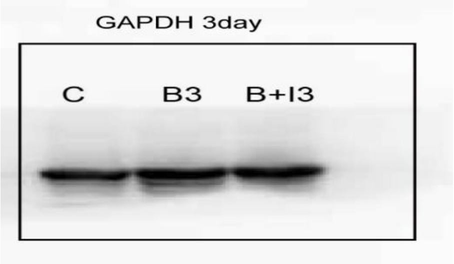

5day

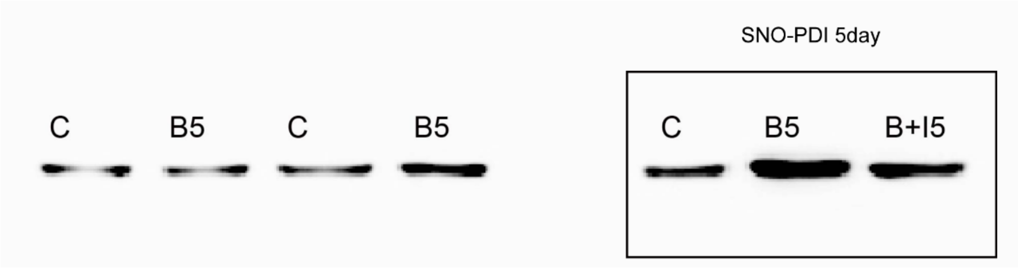
SNO-PDI 5day
C
B5
C
B5
C
B5
B+I5

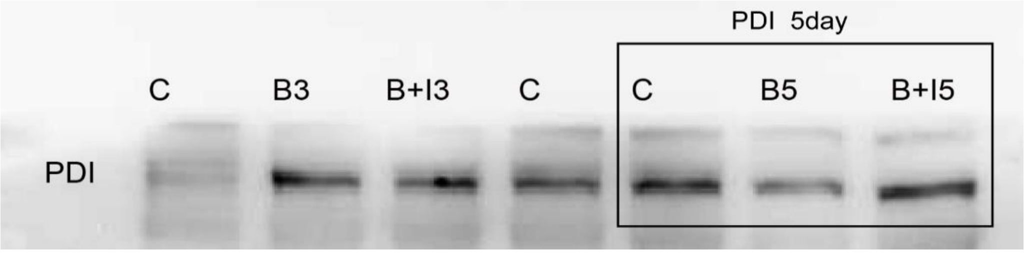
PDI 5day
C
B3
B+I3
C
C
B5
B+I5
PDI

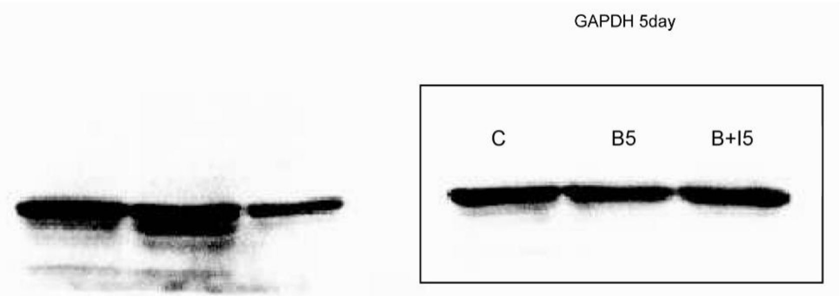
GAPDH 5day
C
B5
B+I5

7day

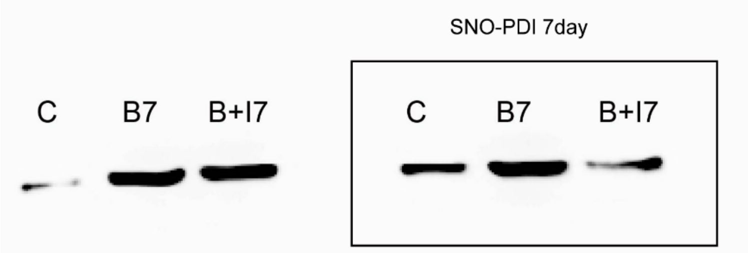
SNO-PDI 7day
C
B7
B+I7
C
B7
B+I7

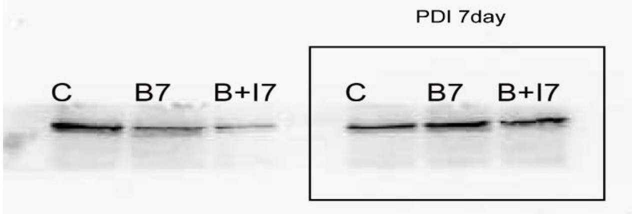
PDI 7day
C
B7
B+I7
C
B7
B+I7

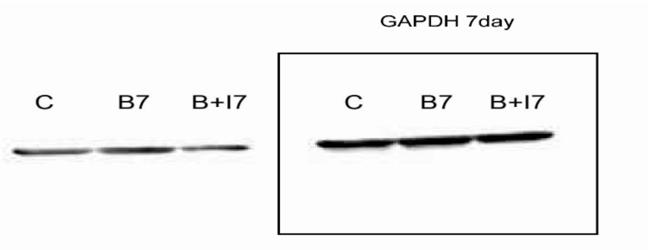
GAPDH 7day
C
B7
B+I7
C
B7
B+I7

**Figure 6.** Effect of ITF on the burn-induced changes in AMPK phosphorylation and autophagy in rat IECs

1 day

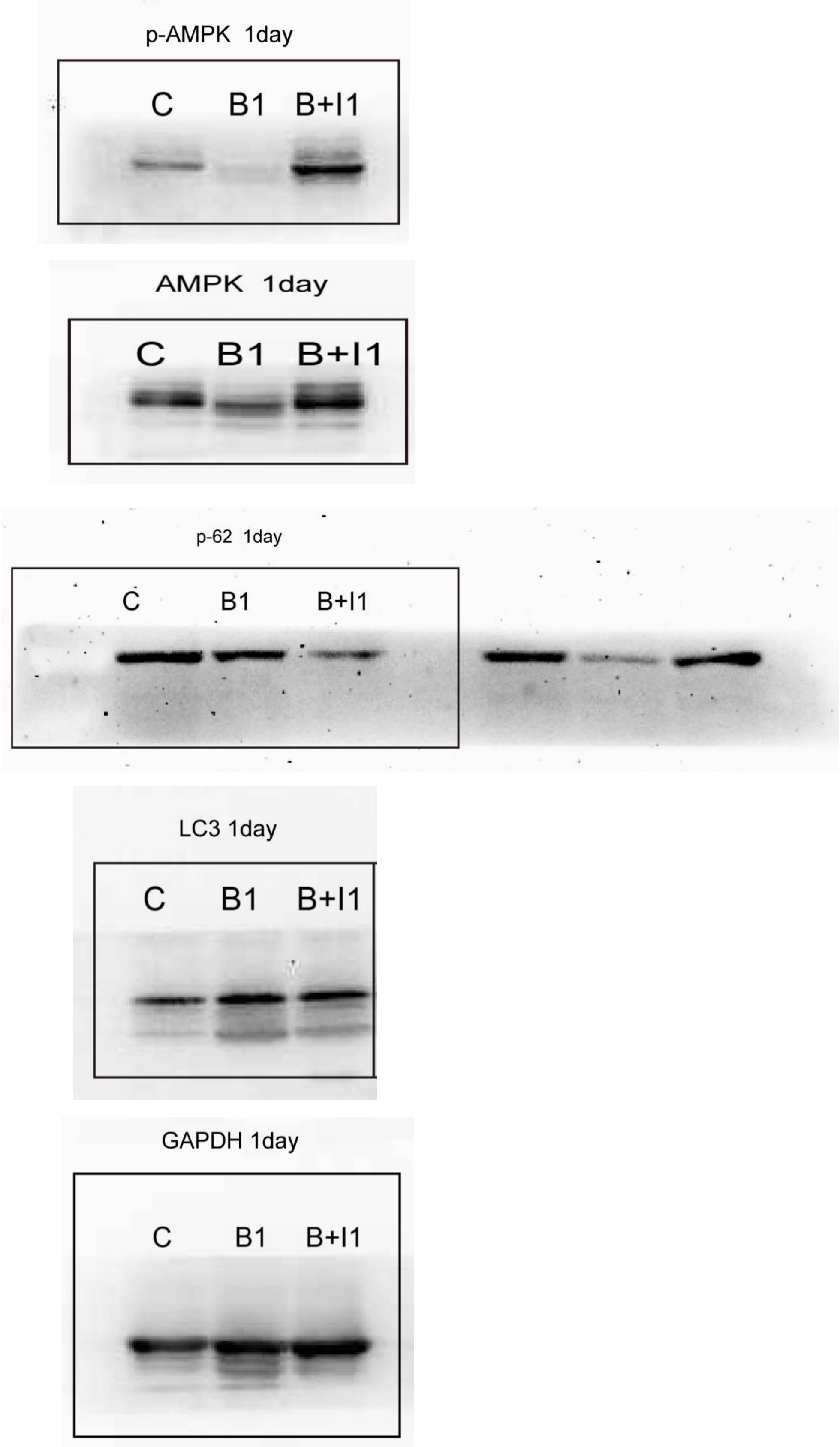

3day

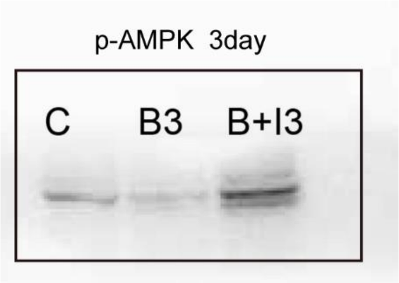
p-AMPK 3day
C
B3
B+I3

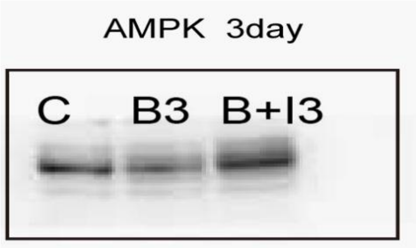
AMPK 3day
C
B3
B+I3

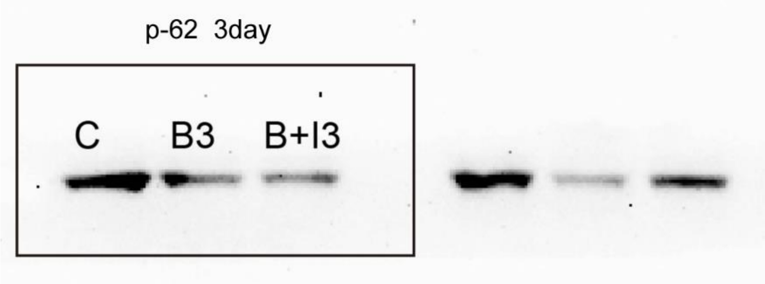
p-62 3day
C
B3
B+I3

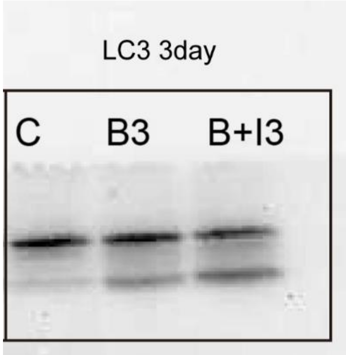
LC3 3day
C
B3
B+I3

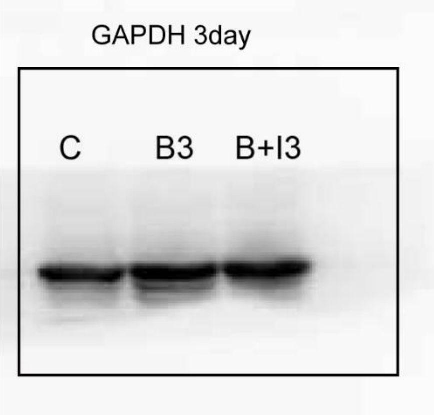
GAPDH 3day
C
B3
B+I3

5day

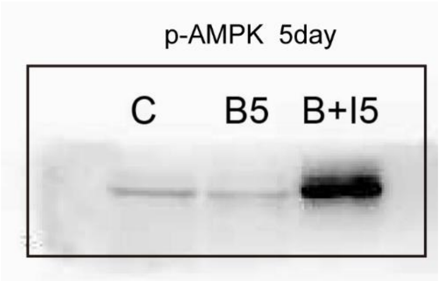
p-AMPK 5day
C
B5
B+I5

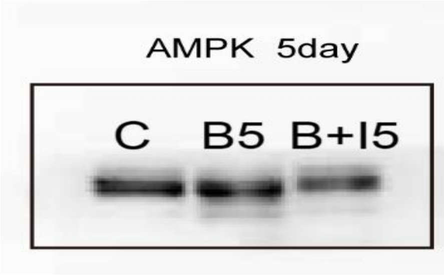
AMPK 5day
C
B5
B+I5

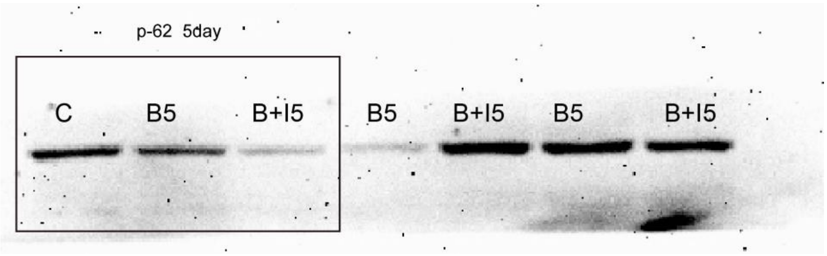
p-62 5day
C
B5
B+I5
B5
B+I5
B5
B+I5

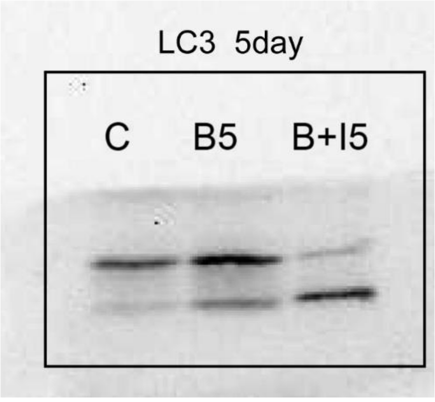
LC3 5day
C
B5
B+I5

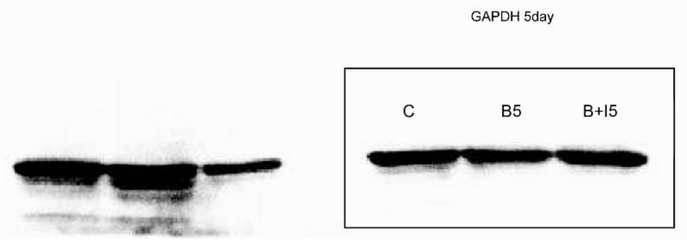
GAPDH 5day
C
B5
B+I5

7day

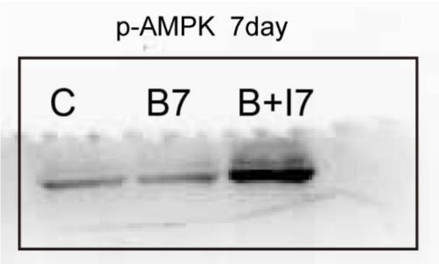
p-AMPK 7day
C
B7
B+I7

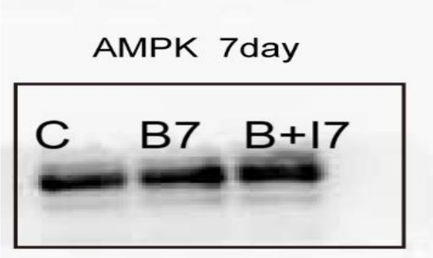
AMPK 7day
C
B7
B+I7

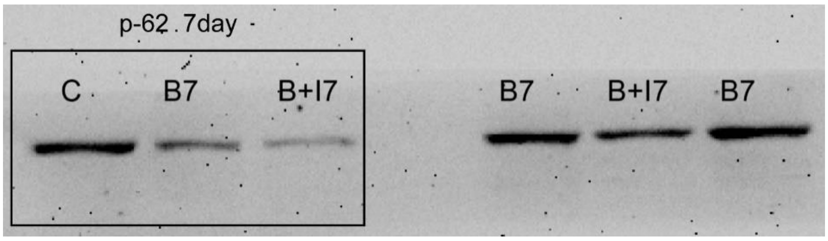
p-62 7day
C
B7
B+I7
B7
B+I7
B7

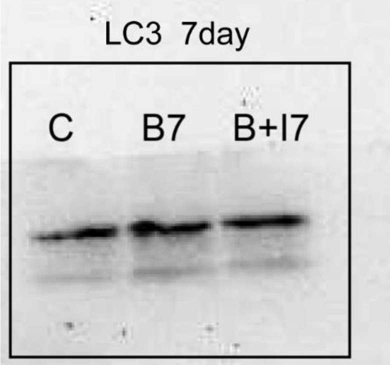
LC3 7day
C
B7
B+I7

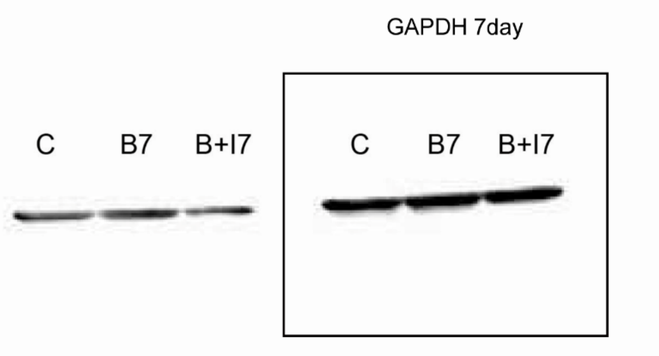
GAPDH 7day
C
B7
B+I7
C
B7
B+I7

**Figure 7.** PDI inactivation on the biological activity of ITF

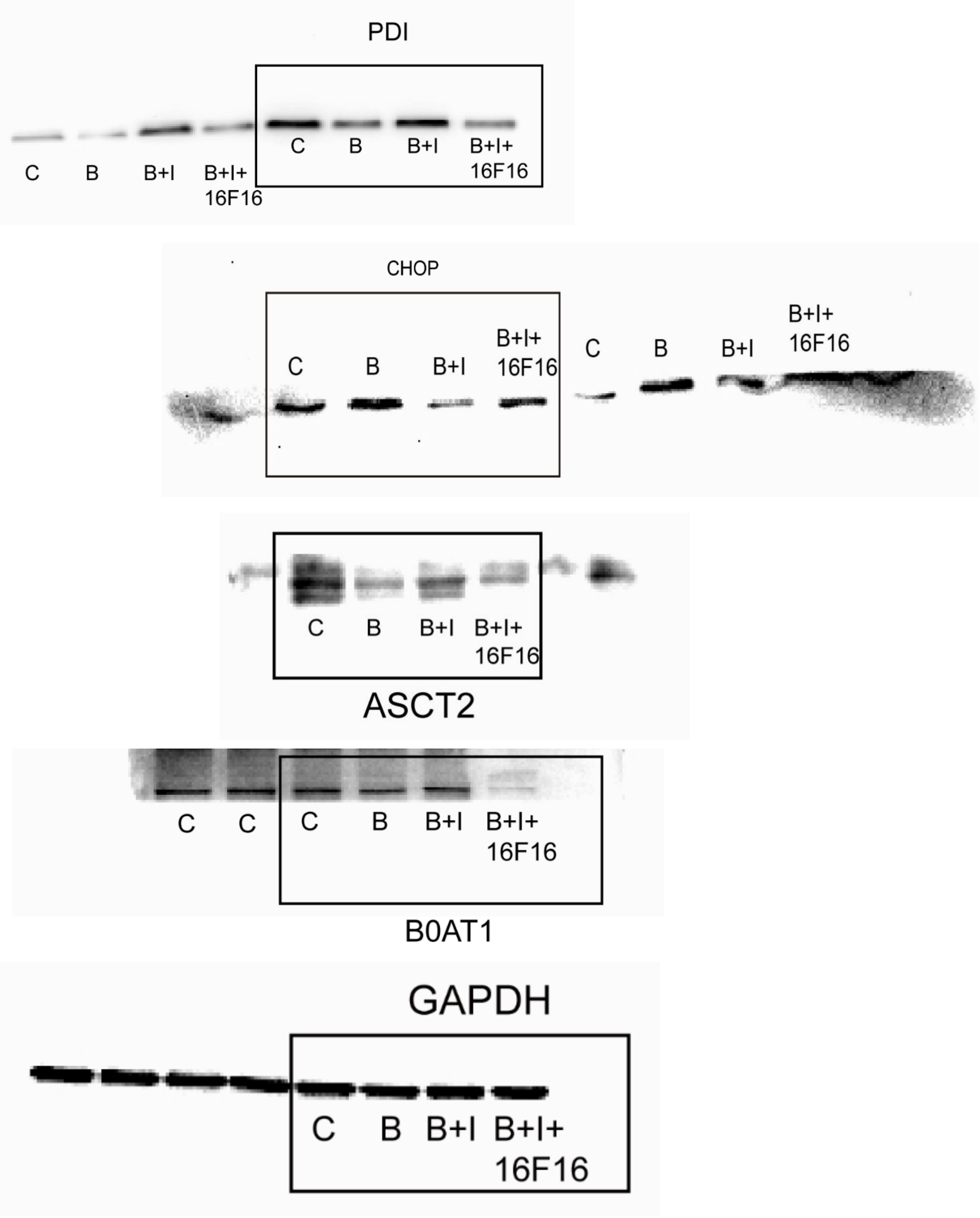

**Figure 8.** Inhibition of autophagy mitigates the biological effect of ITF on IEC-6 cells

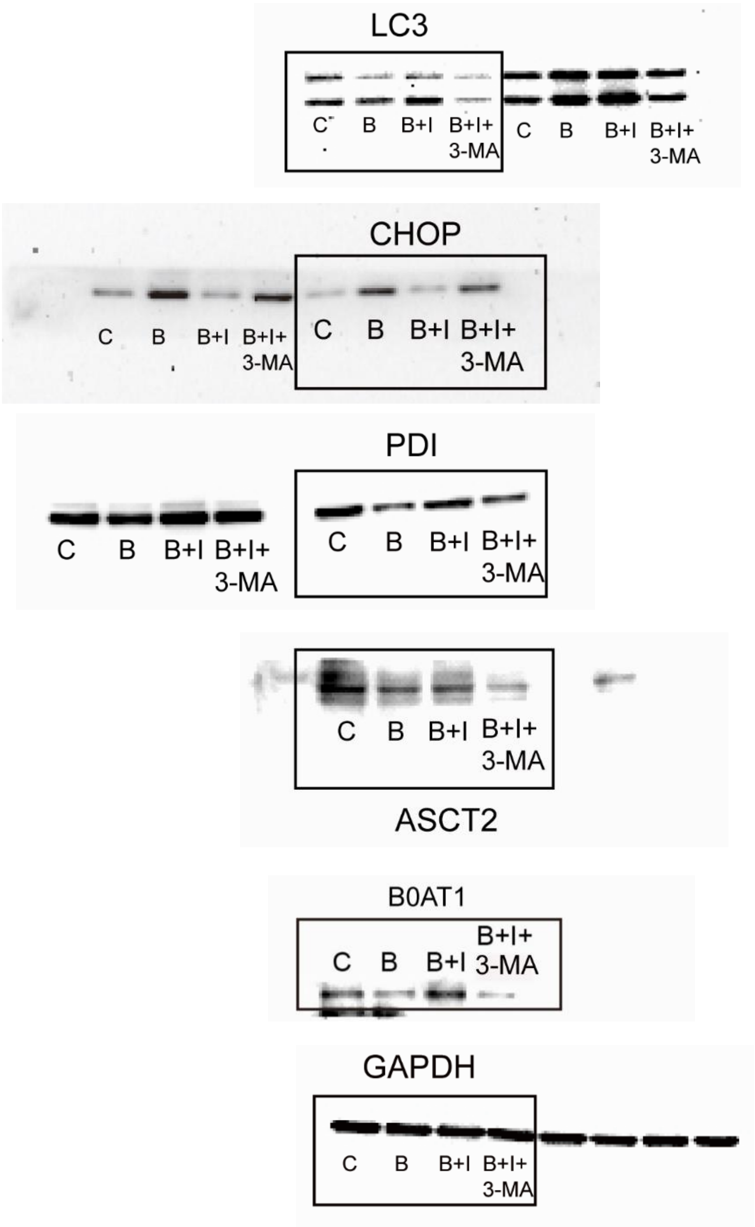

**Figure 9.** Inhibition of AMPK phosphorylation mitigates the biological effect of ITF on IEC-6 cells

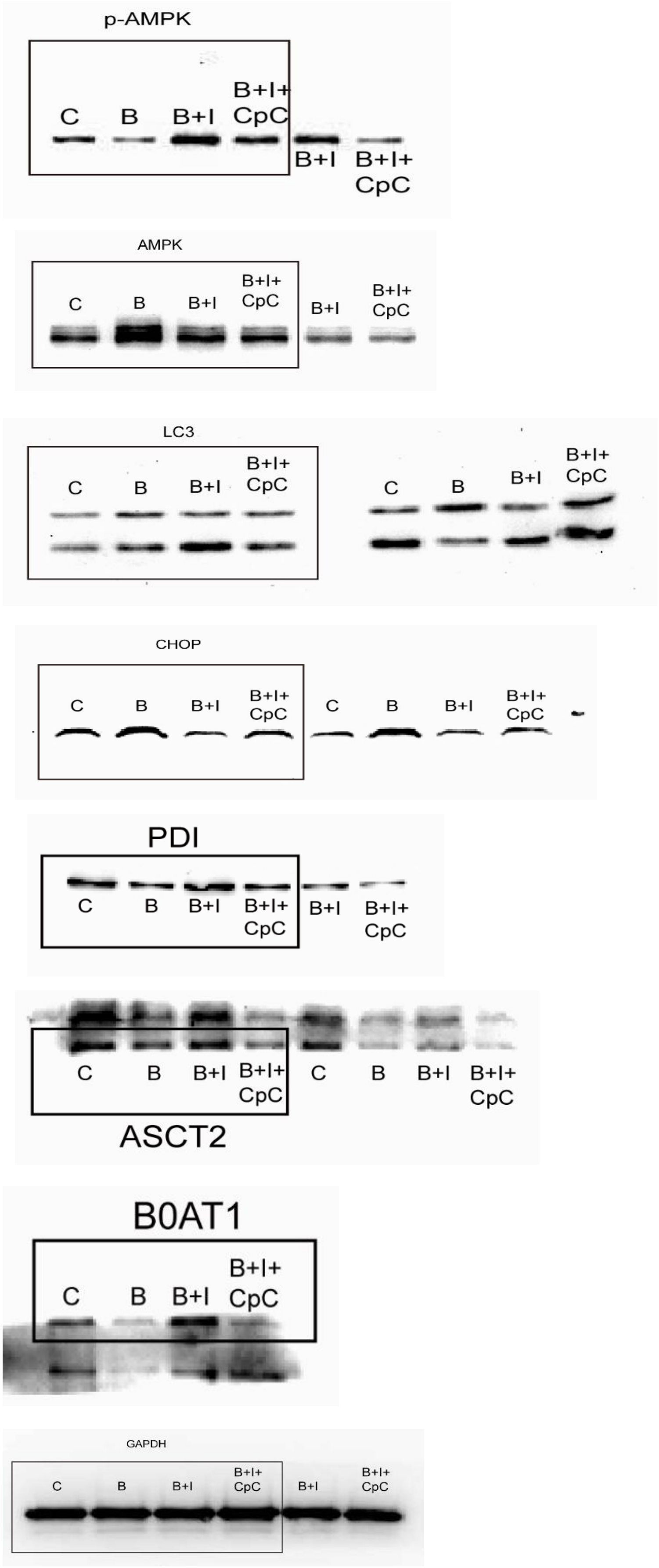

Supplement: Supplementary file 1 — Supplementary Information. [file 41598_2020_69648_MOESM1_ESM.pdf]
